# Diversity of axon initial segment geometry in the mouse hippocampus and its predicted influence on neuronal excitability

**DOI:** 10.1093/cercor/bhaf297

**Published:** 2025-12-09

**Authors:** Nikolas Andreas Stevens, Maximilian Achilles, Juri Monath, Rupert Langer, Maren Engelhardt, Martin Both, Christian Thome

**Affiliations:** Institute of Physiology and Pathophysiology, Heidelberg University, Im Neuenheimer Feld 326, 69120 Heidelberg, Baden-Württemberg, Germany; Institute of Anatomy and Cell Biology, Johannes Kepler University Linz, Krankenhausstrasse 5, 4020 Linz, Upper Austria, Austria; Clinical Research Institute for Neurosciences, Johannes Kepler University Linz, Wagner-Jauregg-Weg 15, 4020 Linz, Upper Austria, Austria; Institute of Anatomy and Cell Biology, Johannes Kepler University Linz, Krankenhausstrasse 5, 4020 Linz, Upper Austria, Austria; Clinical Research Institute for Neurosciences, Johannes Kepler University Linz, Wagner-Jauregg-Weg 15, 4020 Linz, Upper Austria, Austria; Clinical Research Institute for Neurosciences, Johannes Kepler University Linz, Wagner-Jauregg-Weg 15, 4020 Linz, Upper Austria, Austria; Department of Pathology and Molecular Pathology, Kepler University Hospital Linz, Krankenhausstrasse 5, 4020 Linz, Upper Austria, Austria; Institute of Anatomy and Cell Biology, Johannes Kepler University Linz, Krankenhausstrasse 5, 4020 Linz, Upper Austria, Austria; Clinical Research Institute for Neurosciences, Johannes Kepler University Linz, Wagner-Jauregg-Weg 15, 4020 Linz, Upper Austria, Austria; Institute of Physiology and Pathophysiology, Heidelberg University, Im Neuenheimer Feld 326, 69120 Heidelberg, Baden-Württemberg, Germany; Institute of Physiology and Pathophysiology, Heidelberg University, Im Neuenheimer Feld 326, 69120 Heidelberg, Baden-Württemberg, Germany; Institute of Anatomy and Cell Biology, Johannes Kepler University Linz, Krankenhausstrasse 5, 4020 Linz, Upper Austria, Austria; Clinical Research Institute for Neurosciences, Johannes Kepler University Linz, Wagner-Jauregg-Weg 15, 4020 Linz, Upper Austria, Austria

**Keywords:** axon initial segment, axon-carrying dendrite, cell morphology, Hippocampus, pyramidal neuron

## Abstract

Action potentials, the primary information units of the nervous system, are usually generated at the axon initial segment. Changes in the length and position of the axon initial segment are associated with alterations in neuronal excitability, but there is only limited information about the baseline structural variability of this compartment. This work provides a comprehensive analysis of the diversity of proximal cell geometries across all anatomical axes of the murine hippocampus, encompassing dorsal-ventral, superficial-deep, and proximal-distal regions. We analyzed the morphology of 3,681 hippocampal pyramidal neurons in 12 animals of both sexes, focusing on axon initial segment length, position, and association with proximal cellular features such as the soma and apical dendrite. Notably, neurons with axon-carrying dendrites were significantly more common in ventral compared to dorsal hippocampal areas, which we also found in two of three human samples. We employed NEURON simulations to assess the functional implications of this variability. Here, variation in proximal geometry contributed only minimally to neuronal homeostasis, but instead increased heterogeneity of response patterns across neurons.

## Introduction

Neuronal populations are often separated and categorized by common morphological characteristics, such as the geometry of the soma, axonal arborization, or the shape of the dendritic tree. While practical, this approach reinforces a simplified concept of a “typical neuron,” disregarding the substantial diversity within individual regions and subregions. In fact, the “typical neuron” may constitute an exception rather than the rule in a complex and varied neuronal landscape.

The shape of the cell body and proximal neurites has profound impact on how neurons integrate inputs and generate firing patterns (Jiang et al. 2015; Gouwens et al. 2020; Rotterman et al. 2021; Mertens et al. 2024). The axon initial segment (AIS) plays a pivotal role in this context. Due to its high expression of voltage-gated ion channels (Boiko et al. 2003; Kole et al. 2008) and favorable electrotonic conditions, most action potentials (APs) are generated at the distal AIS (Kim et al. 2005; Palmer and Stuart 2006; Meeks and Mennerick 2007; Royeck et al. 2008; Hu et al. 2009b; Foust et al. 2010; Baranauskas et al. 2013). Remodeling of the AIS, as well as mutations in its scaffolding proteins, have been associated with pathologies such as bipolar disorder, epilepsy, intellectual disability, multiple sclerosis, autism spectrum disorder, mouse models of fragile X syndrome, and stroke (Ferreira et al. 2008; Wimmer et al. 2010; Buffington and Rasband 2011; Hsu et al. 2014; Stoler and Fleidervish 2016; Huang and Rasband 2018; Booker et al. 2020; Fujitani et al. 2021; Senol et al. 2022; Usui et al. 2022; Garrido 2023; King et al. 2025). Several studies have demonstrated that the length and composition of the AIS as well as its distance from the soma undergo plastic changes in response to alterations in neuronal activity (Jamann et al. 2018; Freal and Hoogenraad 2025). This AIS plasticity in turn correlates with functional changes in the respective cells (Grubb and Burrone 2010; Kuba 2012; Jamann et al. 2021). Computational studies suggest that the AIS geometry directly impacts neuronal excitability, firing precision, and network behavior (Gulledge and Bravo 2016; Hamada et al. 2016; Kole and Brette 2018; Lazarov et al. 2018; Goaillard et al. 2019; Goethals and Brette 2020), but see also (Moubarak et al. 2019). However, it remains unclear how AIS plasticity relates to the natural variability of initial segments. Longer AIS are associated with elevated neuronal excitability due to their increased area for voltage-gated ion channels, which are critical for triggering APs (Gulledge and Bravo 2016; Jamann et al. 2021; Bakos et al. 2024). The impact of AIS location within the axonal arbor on excitability is multifaceted. Somatic membrane potentials depolarize the proximal AIS with little attenuation. However, the substantial capacitive sink provided by the large somatic membrane is thought to reduce neuronal excitability, rendering distal AIS locations again more excitable than proximal ones (Gulledge and Bravo 2016; Kole and Brette 2018; Goethals and Brette 2020). The situation becomes even more complex when the AIS emerges from a dendritic branch, creating an electrically privileged site for synaptic input (Thome et al. 2014; Hamada et al. 2016; Hofflin et al. 2017). This cell morphology was studied mainly in mouse CA1 pyramidal cells (Thome et al. 2014; Hodapp et al. 2022; Stevens et al. 2024), thick-tufted layer V pyramidal neurons of rats (Hamada et al. 2016), and primary culture (Han et al. 2025), but it was also confirmed in the human hippocampus (30% to 44% in CA1) (Benavides-Piccione et al. 2020) and neocortex (approximately 2%) (Wahle et al. 2022; Benavides-Piccione et al. 2024). Inputs received at these axon-carrying dendrites (AcDs) have privileged access to the AIS, especially during network states in which perisomatic inhibition limits somatic depolarization. Most CA1 pyramidal neurons that fire during sharp wave ripple oscillation in vivo have dendritic axon origins (Hodapp et al. 2022). These cells were also found to receive preferentially contralateral input and have more stable AIS geometry compared to cells with somatic axon origin (Stevens et al. 2024; Han et al. 2025). Electrophysiological recordings have demonstrated that neurons with AcD morphology are indistinguishable from their counterparts in terms of their basic electrophysiological properties when recorded at the soma (Thome et al. 2014; Hamada et al. 2016; Stevens et al. 2024). It is thus conceivable that variations in AIS length and positions are counteracted by other morphological parameters such as the size of the soma or apical dendrite (Gulledge and Bravo 2016). In cortical AcD neurons, the diameter of the apical dendrite decreases with AIS distance, thereby normalizing its impact on the AP threshold and waveform (Hamada et al. 2016).

The rodent hippocampus is segregated anatomically, genetically, and functionally into the ventral, intermediate, and dorsal portions along its longitudinal axis (Bannerman et al. 2004; Strange et al. 2014). Functionally, the dorsal and medial sections of the hippocampus are preferentially involved in spatial learning and the ventral part plays an important role in mediating anxiety-like behavior and emotional learning (O'Keefe and Dostrovsky 1971; Moser and Moser 1998; Richmond et al. 1999; Kjelstrup et al. 2002). Place fields are more discrete and precise in the dorsal end of the hippocampus, gradually becoming larger towards the ventral end (Jung et al. 1994; Kjelstrup et al. 2008; Royer et al. 2010). The dorsal hippocampus receives visuospatial information via the medial entorhinal cortex, whereas the ventral hippocampus is highly connected to the amygdala, hypothalamus, and sensory areas (Amaral and Witter 1989; Cenquizca and Swanson 2007; Fanselow and Dong 2010). The intermediate hippocampus receives input from visual, prefrontal, and subcortical areas including the amygdala and hypothalamus (Fanselow and Dong 2010). This overlapping connectivity may allow this region to play a specific role in rapid place learning tasks (Bast et al. 2009). Disease-related activities also differ between hippocampal sections. Epileptic seizures were found to originate mainly from the ventral and not the dorsal hippocampus (Racine et al. 1977; Akaike et al. 2001; Toyoda et al. 2013). In contrast, dorsal CA1 pyramidal cells are more prone to damage by ischemia (Ashton et al. 1989). Gene expression studies in rodents also provided evidence for distinct molecular boundaries within the dorsal-ventral axis of the hippocampus (Thompson et al. 2008; Dong et al. 2009; Fanselow and Dong 2010; O'Reilly et al. 2015). Ex vivo studies using hippocampal slices showed that CA1 pyramidal neurons gradually increase in excitability from the dorsal to the ventral end of the hippocampus (Dougherty et al. 2012; Malik et al. 2016; Milior et al. 2016). This enhanced excitability towards ventral CA1 encompasses rising firing frequencies, input resistances, AP thresholds, and resting membrane potentials. At the same time, CA1 neurons in the ventral part have fewer dendritic branches and less dendritic surface area compared to dorsal CA1 neurons. The electrophysiological differences are further accompanied by differential expression of voltage-gated ion channels, such as HCN channels (Marcelin et al. 2012; Dougherty et al. 2013), which play a crucial role in maintaining the resting properties of CA1 neurons and significantly affect the integration and transfer of voltage signals along the somatodendritic axis (Vaidya and Johnston 2013).

The present study investigates AIS morphologies along the primary anatomical axes of the hippocampus: transverse (proximo-distal), radial (deep-superficial), and longitudinal (dorsal-ventral). Given the well-characterized differences between these locations in terms of behavioral roles, anatomical connectivity, electrophysiology, and gene expression, it is plausible that populations of pyramidal neurons may exhibit distinct AIS morphologies tailored to the computational demands of their regions.

## Materials and methods

### Animals and sample preparation

Animals were housed at the Interfaculty Biomedical Facility (IBF) at Heidelberg University and approved by the local animal welfare officer. All procedures were performed in strict accordance with the guidelines of the European Community Council and the state government of Baden-Wurttemberg. We used male and female individuals of the Thy1-GFP mouse line M aged 3 to 4 months (Strain #: 007788; RRID: IMSR_JAX:007788, (Feng et al. 2000)). Animals were anesthetized with CO_2_ and decapitated after the loss of the righting reflex. The brain was quickly removed and transferred into ice-cold (12 °C) artificial cerebrospinal fluid (ACSF) containing the following (in mM): 124 NaCl, three KCl, 1.8 MgSO_4_, 1.6 CaCl_2_, 10 glucose, 1.25 NaH_2_PO_4_, saturated with 95% O_2_/5% CO_2_ and calibrated to pH 7.4. The cerebellum and the first third of the frontal brain were removed, and 350 μm horizontal slices were cut using a vibrating blade microtome (Leica VT1000S, Leica Biosystems, RRID: SCR_016495) for medial and ventral sections (~750 μm apart). Coronal slices were made for dorsal hippocampal sections.

Immunofluorescence and confocal imaging: slices were fixed in 2% paraformaldehyde (PFA; Sigma-Aldrich) diluted in 0.1 M phosphate buffer, pH 7.4, at room temperature for 90 min. After fixation, slices were stored in phosphate-buffered saline (PBS; in mM, 137 NaCl, 2.7 KCl, eight NaH_2_PO_4_, pH 7.2) at 4 °C for up to 1 week. AIS were visualized using the primary antibody rabbit-anti-βIV-spectrin (1:1000; self-made, corresponding to amino acids 2237 to 2256 of human βIV-spectrin; (Gutzmann et al. 2014)) combined with secondary antibody Alexa 647 anti-rabbit 1:1000 (Molecular Probes, RRID AB_2536183). The staining procedure was performed as follows: Slices were washed, blocked for 2 h in blocking solution (5% normal goat serum from Vector Laboratories, RRID:AB_2336615, 0.5% Triton X-100 from Merck (CAS: 9036-19-5) in PBS); washed again and incubated in primary antibody solution (0.2% Triton X-100 and 1% NGS in PBS) overnight at room temperature. Slices were again washed and incubated for 2 h in the secondary antibody solution. All washing steps were repeated three times for 15 min in PBS and incubation steps performed on an orbital shaker. After the final washing step, slices were mounted on microscope slides using Fluoroshield (Abcam, Danaher Corporation, ab104139) with DAPI to visualize the hippocampal cell band. Slices were imaged with a Nikon A1R confocal microscope (RRID: SCR_020317). We used objectives with magnification factors 10× (Nikon Plan Apo λ 10× NA 0.45) for overviews and 60x (Nikon N Apo 60× NA 1.4 λ oil immersion) for confocal stacks of 0.5 μm z resolution to capture cell morphologies. Human samples were imaged using a Leica Stellaris 5 confocal microscope (RRID:SCR_024663) with 10× (Leica HC PL Fluotar 10×/0.30) and 63× objectives (Leica HC PL APO 63x/1.30 GLYC CORR, glycerin immersion). Images were analyzed in 3D using Fiji/ImageJ (RRID:SCR_002285; Wayne Rasband, NIH) for human and simple neurite tracer package (RRID:SCR_014146)(Arshadi et al. 2021) for mouse samples.

### Anatomical segmentation

**Fig. 1:**
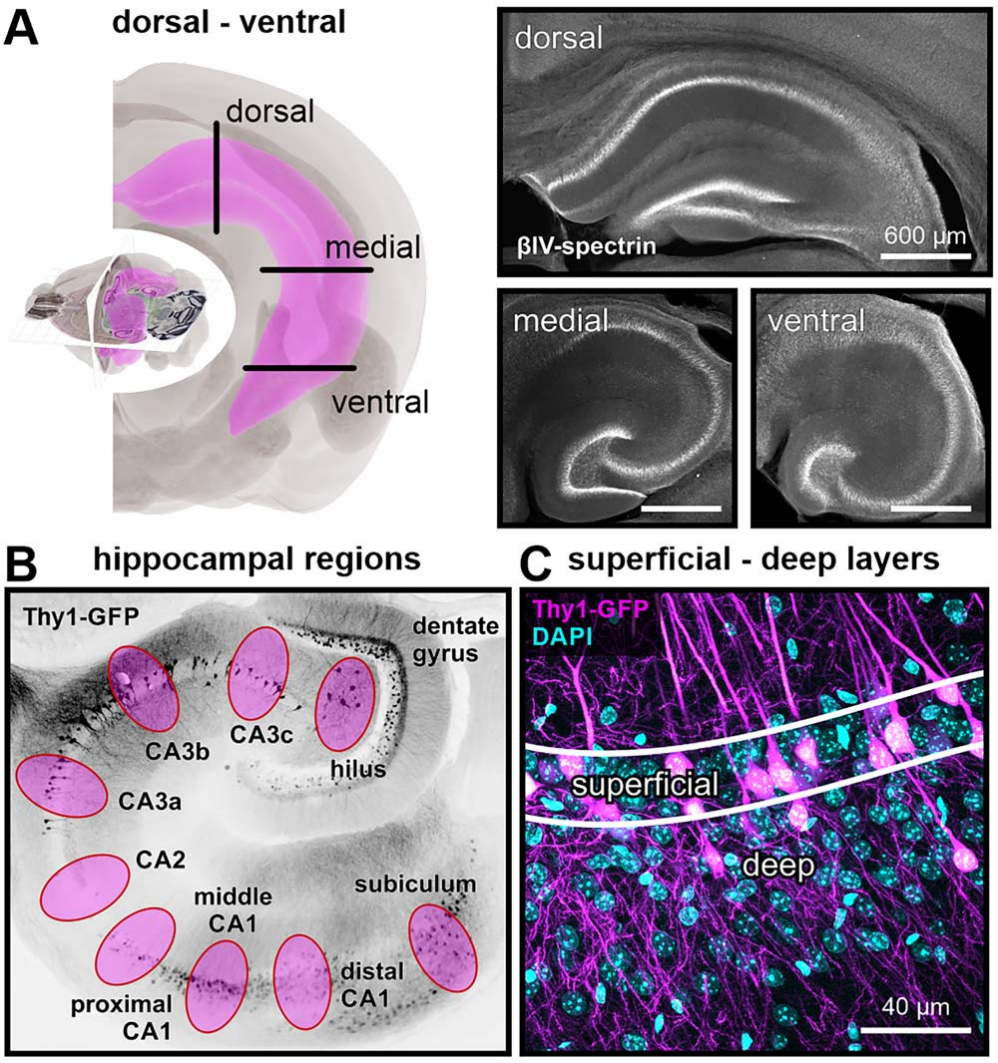
Anatomical axes in the mouse hippocampus. a) The left panel was modified from 3D Brain Explorer (2018 Allen Institute for Brain Science) and shows the longitudinal orientation of the hippocampal formation in the murine brain. The right panel shows confocal images of AIS labeling along the pyramidal cell band (βIV-spectrin signal) in brain slices obtained in the orientation visualized on the left. b) Confocal image of Thy1-GFP signal in a ventral hippocampal slice. Hippocampal subregions used for analysis are marked. c) Confocal image of Thy1-GFP and DAPI signals illustrating the separation of the dense ~2 cell bodies wide superficial layer from the deep layer.

Slices used for morphological analysis along the dorsoventral axis were selected based on the following anatomical landmarks identified after immunofluorescent labeling. Ventral hippocampal slices were defined as the first sections in which all hippocampal regions (dentate gyrus, CA fields, subiculum) were clearly visible, typically showing a semi-circular dentate gyrus and a smooth, continuous pyramidal cell band. Samples for the dorsal hippocampus were selected for having a horizontal dentate gyrus and CA3c base. Anatomical subregions were defined according to cell morphology, location, and orientation as follows: Hilus: disorganized cells between dentate gyrus blades; CA3c: pyramidal cells in the first 30 μm of the CA3 cell band; CA3b: cells in the curvature of the band; CA3a: cells at the end of CA3 but outside CA2; CA2: position in cell band and lack of GFP signal (confirmed by other labs in personal communication). We verified the CA2 position using the marker PCP4, which consistently labeled neurons around the border of stratum lucidum (Stevens et al. 2024). CA1 was split into proximal (start), central, and distal before transition to subiculum. Confocal images were taken in the center of each defined subregion without overlap. We aimed to capture 20 fully analyzed cell morphologies per subregion. If cell numbers were not matched with the initial four animals, additional animals were used. This applied especially for CA3 areas in the dorsal planes. Superficial and deep cells were defined post hoc by their geometric distance towards the stratum radiatum border. Superficial layers were defined as ~2 neurons wide and considering the total thickness of the cell band (30 to 50 μm; Fig. 1; Supplemental Fig. S1A and B).

### Cell classification and tracing

Soma size was defined as convex curvature around the Thy1-GFP label. Cell size was captured as an ellipse defined by the two longest orthogonal diameters. The thickness of apical dendrites was captured by five consecutive diameters starting from the base of the somatic circumference with 5 μm spacing. We initially used automated approaches using thresholds in fluorescence intensities to define the start and end of initial segments, but these were error-prone due to high overlap in our dataset. Therefore, the proximal beginning of the AIS was determined by the first clearly visible immunoreactive segment along the axon, marked by a strong increase in the βIV-spectrin fluorescence. The AIS end was defined as the last point of consecutive labeling along the axon. AIS diameter was captured by diameters at the AIS start, end, and point of maximum width, as well as distance from AIS start to point of maximum width. In cases where the axon was connected to a somatic protrusion that also gave rise to a dendrite, we measured the length and diameter of this segment (AcD stem). Cells were classified as AcD cells during post hoc data processing when the potential AcD stem was at least 2 μm long and longer than its mean diameter.

### Human tissue samples

Human hippocampal tissue was obtained from deceased donors through the Department of Pathology at the Johannes Kepler University Hospital Linz, in accordance with Austrian law (Krankenanstaltengesetz §25) and positive ethics approval evaluation (see statement below). Hippocampal samples were collected from five donors, but due to postmortem delay and staining quality, only three cases were selected for further morphological analysis. Donor information is summarized in the following order: patient-ID, age, sex, and postmortem delay. Patient-1/56/M/22 h. Patient-2/62/M/5 h. Patient-3/76/F/7 h. There was no known neurological disease history for any of the three patients. During autopsy, a tissue block containing the hippocampal formation and adjacent cortical areas was excised. Each hippocampus was divided into three blocks along the rostro-caudal axis (rostral, central, and caudal), and stored in 1× PBS on crushed ice until further processing. Rostral blocks used for analysis excluded the very anterior tip of the hippocampus and began at the first coronal level in which the subiculum was visible. The sagittal position was confirmed using anatomical literature (Donkelaar 2018). Sections were cut at a thickness of 300 μm using a vibratome, and processed identically to mouse tissue, except that fish gelatin was used instead of NGS. Neurons were labeled using a polyclonal chicken anti-NeuN antibody (Synaptic Systems, RRID: AB_2571734) in conjunction with a secondary anti-chicken-488 (ThermoFisher, RRID: AB_2534096). For AIS labeling see sections above. The staining was verified using cortical tissue from pig brains (*Sus scrofa domesticus*) as positive control (48 h postmortem, from a local slaughterhouse) and the respective human tissue as negative controls omitting the primary antibodies.

### Ethics approval disclosure statement

The study was approved by the Ethics Committee of the Medical Faculty at the Johannes Kepler University Linz (Approval No.: 1379/2024).

### Computer simulations

Computer simulations were carried out using the NEURON simulation environment through its python module (Hines et al. 2009). Cell morphology, parameters and synaptic stimulation paradigms are reused and adjusted for AIS geometry from (Hodapp et al. 2022) available under https://doi.org/10.11588/data/JWLFFZ. Morphological parameters of the original model were modified according to our measurements, namely: soma size (3D surface of calculated ellipsoid), AIS distance from the soma, AIS length, AIS diameter (proximal and distal parts as well as location of largest diameter), proximal apical dendrite diameters at five locations (5, 10, 15, 20, and 25 μm from the soma), and AcD stem dendrite length and diameter for AcD cells. For the simulations, time step duration was set to 25 μs (corresponding to 40 kHz). The soma gave rise to one apical dendrite and two basal dendrites. The apical dendrite was divided into three parts with different diameters and ion channel composition and terminated into two distal dendritic branches mimicking an apical tuft (biophysical properties given in Supplemental Figures). One of the basal dendrites was connected to the AIS (AcD branch), while the other (non-AcD branch) carried another dendritic structure. This segment was shifted along the non-AcD dendrite in parallel to the axon distance to achieve electrotonic symmetry between AcD and non-AcD branches. The AIS was constructed out of two parts with the activation threshold of Na^+^ channels in the distal part shifted by −5 mV to simulate experimental findings (Hu et al. 2009b). Segmentation around the dendritic injection sites was made with high resolution (0.5 μm) to keep high-frequency terms of the input signal. Active conductances were modified from previously reported values (Hu et al. 2009a; Cutsuridis et al. 2010; Thome et al. 2014; Hodapp et al. 2022). We limited our analysis to two basal dendrites, given the low number of basal dendrites in hippocampal pyramidal cells (Hodapp et al. 2022; Stevens et al. 2024) and our specific interest in comparing the AcD branch with a canonical basal dendrite. Stimulation was achieved either via conductance-based synaptic activation or direct current injection. For synaptic stimulation, 1000 synapses (reversal potential: 0 mV; τ_on: 0.175 ms; τ_off: 5 ms) were evenly distributed 75 to 100 μm from the soma on either the axon-carrying or canonical basal dendrite. Synaptic activation times followed a Gaussian distribution (σ = 4 ms), with onset beginning 20 ms after simulation start. The high synapse count ensured sufficient resolution to detect subtle differences in synaptic transmission. Total synaptic conductance ranged from 1 to 100 nS. For current injection simulations, a constant current was applied for 5 ms, starting 20 ms after simulation onset. Injection sites included the soma, center of proximal AIS, or center of distal AIS. Current amplitudes ranged from 10 to 1000 pA. Synaptic strength or current amplitude was incrementally increased until an AP was generated, defined as the membrane potential at the AIS exceeding 0 mV. The minimal stimulus required to evoke an AP was recorded and two waveform parameters were quantified: (i) AP voltage threshold, defined as the membrane potential where the rate of change exceeded 25 mV/ms at the soma, and (ii) AP delay, defined as the time between stimulation onset and threshold crossing.

### Statistical methods

Cell morphologies were collected from up to 20 individual cells per subregion per hippocampus. The resulting distributions exhibited either symmetrical bell-shaped or log-normal characteristics (eg AcD distance, AcD stem length). Log-normal distributions were logarithmically transformed before applying statistical tests such as ANOVA or correlation analysis. Statistical significance was set at *P* < 0.05 for all tests, with significant *P*-values denoted in figures by asterisks (< 0.05*, < 0.01**, < 0.001***). All analyses were conducted using the Python module Pingouin (RRID:SCR_022261)(Vallat 2018). One statistical limitation warrants attention: Cells and hippocampi were treated as independent biological replicates in correlational analyses. We aimed to sample 20 fully characterized pyramidal cells per location within each anatomical subregion. In instances of low GFP expression, additional animals were included to achieve approximately equal cell counts across subregions. Notably, medial and ventral samples were derived from the same animals, while dorsal slices were prepared from separate individuals. To assess the influence of sex, hemisphere, and animal on cell geometry parameters, we performed 3-way ANOVAs, finding no significant differences between classes. Consequently, values from each hippocampal location with more than nine fully measured cells are represented in Figures as white individual data points. While we consider this approach suitable for descriptive purposes and controlled for potential confounds using partial correlation tests, it may affect the precision of statistical tests used in the second Figure (comparison of hippocampal planes and main areas). For further reference, animal identifiers, sex, and hemisphere information are included in the dataset. The R2 value was used to assess the strength of the correlations between morphological parameters and anatomical location. Specifically, it quantifies the proportion of variance in one variable that can be predicted by another.

To assess and compare the variability of six morphological parameters across different hippocampal regions, we first applied a log transformation to AIS distance and AcD stem length to address their non-normal distribution. Subsequently, we normalized all parameters globally by calculating Z-scores across the entire dataset. Variability was then computed for each hippocampus as the standard deviation of the Z-scored values within each hippocampal region and anatomical axis. The variability data was visualized using either line plots with confidence intervals based on the standard deviation of variability across individuals or letter-value plots. These extended box plots (Letter-value plots, boxenplots in pythons seaborn package; RRID:SCR_018132) show distribution of AIS length, AIS distances, somatic area, and mean apical dendrite diameter between area CA1, CA3, and subiculum. White dots depict median values of the region for each hippocampus. Gray dots and lines depict mean value of all hippocampi pooled with 95% confidence interval. The black dots depict outliers as defined by data points falling beyond the extended ranges determined by the letter-value plot method. Specifically, these ranges are dynamically calculated based on the interquartile range (IQR) at progressively smaller proportions of the data distribution, following the “k_depth” parameter set to “trustworthy.” Outliers represent values that deviate significantly from the main distribution.

## Results

We assessed the proximal cell geometry of hippocampal neurons using the intrinsic fluorescence signal of the Thy1-GFP mouse line, in which a sparse subset of mature projection neurons is labeled with GFP (Morris and Grosveld 1989; Feng et al. 2000). Regions of interest were defined along the main anatomical axes of the hippocampal formation: (i) the dorsal, medial, and ventral hippocampal sections (Fig. 1a), (ii) the hippocampal cell band, divided into the hilus, CA3c, CA3b, CA3a, proximal CA1 (CA1p), central CA1 (CA1m), distal CA1 (CA1d), and subiculum (Fig. 1b), (iii) along the superficial to deep axis (Fig. 1c). We traced ~160 cells per subregion in CA1 and CA3, and subiculum, 12 animals, 3 to 4 months old, both sexes (see Supplemental Fig. S1). Of note, the Thy1-GFP line used in this study (M-line) shows no GFP signal in CA2, thus only AIS length measurements were taken here. Since the hilar Thy1-positive cell population is mostly comprised of mossy cells that are morphologically and functionally distinct from pyramidal neurons, we provide them as a resource in our published dataset, but have omitted them otherwise.

**Fig. 2:**
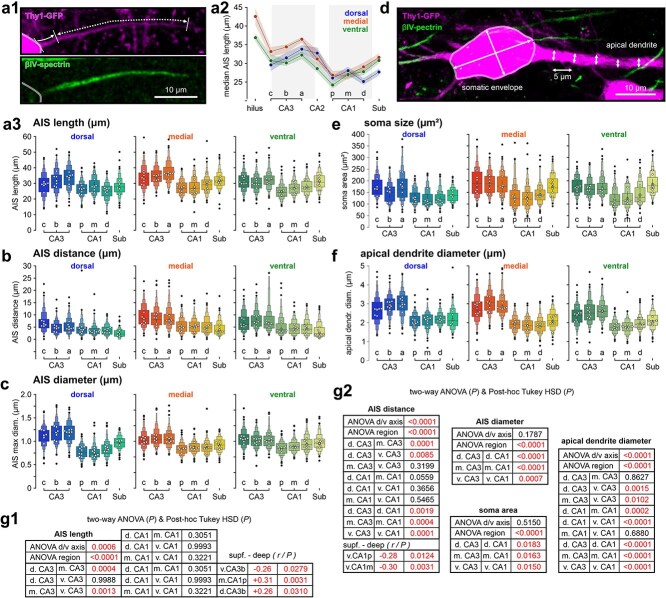
Proximal cell geometry in hippocampal subregions. a1) Confocal image of intrinsic GFP label (magenta) and immunolabeled AIS (βIV-spectrin). Arrows show length and distance of βIV-spectrin signal within the axon. a2) Length distribution of AIS across the hippocampal cell band of the dorsal, medial, and ventral hippocampal formation. a3) Extended box plots visualize length distributions of the AIS across the different subregions of the hippocampal formation. Median values given in white, mean of medians with SD in gray, and computed outliers in black (see methods for details on letter-value plots, some outliers, fewer than 10 outliers across Fig. 2 and 3 are omitted for clarity). b and c) Plots visualize the distribution of AIS distance from the soma b) and maximum diameter across different subregions of the hippocampal formation c). d) Confocal image of intrinsic GFP label and immunolabeled AIS (βIV-spectrin). Soma size was captured as an approximated ellipse by measuring the longitudinal length and diameter of the soma. Apical dendrite diameter as mean from five consecutive width measurements with 5 μm spacing. e and f) Extended box plots visualize the distribution of soma size, simplified as ellipse area e), and mean apical dendrite diameter f) across hippocampal subregions. g1 and g2) Statistical comparison between hippocampal areas. *P* values are derived from two-way ANOVA tests, followed by Tukey post hoc analysis. Correlations across the superficial to deep axes were analyzed using Spearman correlation followed by Bonferroni correction across all subregions (*n* = 12 animals, 23 hippocampi, 2880 cells).

**Fig. 3:**
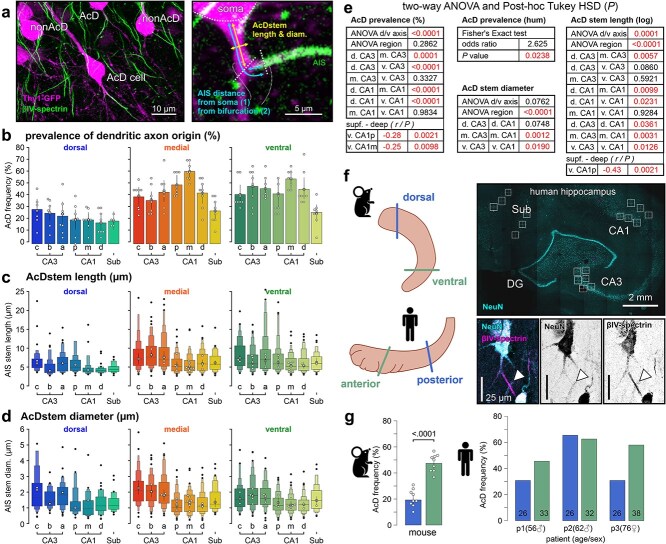
Dendritic axon origins are commonplace in the hippocampal formation. a) Confocal image of pyramidal neuron with dendritic axon origin (AcD cell). Cells were classified as AcD cell if the segment separating soma from axon (AcD stem) was >2 μm long and longer than its diameter (AcD stem diameter). b) Bar plots illustrating the mean percentage of AcD cells among all cells in the respective region. Markers as in Fig. 2. c and d) Extended box plots of AcD stem lengths and diameters between different hippocampal subregions (plot details given in statistical methods). e) Statistical comparison between hippocampal areas and sections across the dorsal to ventral axes of the hippocampus. *P* values are derived from two-way ANOVA tests, followed by Tukey post hoc analysis. f) Left panel: orientation of homolog sections of the human and mouse hippocampus. Right panel: confocal images of hippocampal formation from a human sample with CA1 neuron (bottom) visualized by staining against NeuN and βIV-spectrin. g) Ratio of AcD to non-AcD cells in ventral/anterior and dorsal/posterior cutting sections of eight mice (left) and three humans (right) of different age and sex.

We measured the size of the soma, the diameter of the proximal portions of the apical dendrite, the length, diameters, and position of the AIS, and in the case of dendritic axon onsets, the length and diameter of the AcD stem dendrite separating soma and axon-dendrite bifurcation (see confocal images in Fig. 2 and Fig. 3). Our experimental outline was not designed to study the impact of sex or brain hemisphere, but when we compared the median values for each parameter in each hippocampal location, we found no significant differences between those groups. Since we focused our study on intrinsic variability and interactions, we collected an equal number of geometries across all anatomical subregions (20 cells) and pooled them for further analysis.

### Axon initial segment length, distance, and diameter

The location and length of AIS were mapped throughout the hippocampus using immunofluorescence against the scaffolding protein βIV-spectrin (Fig. 2a, Fig. S1C). Initial segments were longest in the hilus region. Along the pyramidal cell band, median AIS lengths increased in the CA3 axis until they reached the highest values in CA3a. Their length then dropped to the lowest at proximal CA1, with CA2 pyramidal cells showing intermediate levels. Across the dorsal to ventral axis, the AIS were longest in the intermediate portion of the hippocampus. Along the superficial to deep axis, AIS were longer in the deeper layers, most prominently in dorsal CA3b and the proximal portion of medial CA1 (*r*-value: +0.26 and + 0.31, respectively).

AIS distance was defined as the spatial separation of the soma and the start of the βIV-spectrin signal (Fig. 2a1–3, Fig. S1). Dendritic AIS origins were included in this analysis (compare Supplemental Fig. S2). The AIS label often started at a considerable distance from the soma across all hippocampal regions (Fig. 2b). AIS distances generally decreased from CA3 towards the subiculum in all hippocampal planes. The most significant reduction was again seen from CA3a to CA1p, where AIS onsets were about 2 μm shorter. AIS distances were largest in the medial hippocampus and shortest in the dorsal planes. Across the superficial to deep axis, the AIS onset moved closer towards the cell body, most prominently in ventral CA1 in the middle and proximal portions (*r*-value: −0.30 and −0.28, respectively). AIS diameters are rarely reported in confocal studies due to limited precision of optical measurements. We captured AIS width by measuring diameters at the AIS start, end, and point of maximum diameter, as well as position of maximum diameter along the AIS (Fig. 2c, Supplemental Fig. S3). AIS maximum diameters increased only moderately within the first 2 to 3 μm of their length, after which they became increasingly thinner. Thus, AIS start diameter and maximum diameters were similar in size. Their distribution across hippocampal regions followed the pattern seen for AIS lengths. Diameters at the end of the AIS were similar between cell groups (Supplemental Fig. S3). Although measurements ~0.5 μm have limited precision in confocal microscopy, our data is in accordance with other reports of minimum AIS diameters (Hofflin et al. 2017; Bonnevie et al. 2020). Since computational studies suggest that the distal end of the AIS is a better predictor of neuronal excitability than its proximal start or total length (Goethals and Brette 2020), we also compared the distal end of the AIS between hippocampal pyramidal cells across all anatomical axes (Supplemental Fig. S4). The distribution of AIS endpoints (AIS distance + AIS length) followed the pattern observed for AIS lengths very closely. AIS length and distance correlated negatively in CA3 and subicular neurons of medial and ventral planes (medial CA3: *P* < 0.001, medial subiculum: *P* = 0.0035, ventral CA3: *P* = 0.0005, ventral subiculum: *P* = 0.0818, pairwise Spearman correlation). This demonstrates that distal AIS location may be partially compensated by shorter AIS.

### Soma and apical dendrite

The somatic membrane, combined with the proximal apical dendrite, provides a considerable sink for synaptic currents, which renders cell size a strong predictor of neuronal excitability (measured as rheobase). However, it also interacts directly with spike initiation at the AIS through electric interactions such as resistive coupling (Gulledge and Bravo 2016; Goethals and Brette 2020). Somata were smaller in the dorsal compared to the medial and ventral hippocampus (Fig. 2d and e), but the largest differences occurred between hippocampal areas within each cutting plane. Somata of CA3 neurons were generally larger than those of CA1 neurons, while subicular neurons show mid-range levels in dorsal and CA3-like levels in medial and ventral planes. The diameter of the proximal apical dendrite was visualized as the mean of five consecutive diameters with 5 μm spacing (Fig. 2f), with the distribution mirroring the pattern found for soma size. Apical dendrites in the dorsal hippocampus were thinner compared to their medial and ventral counterparts. The main hippocampal areas showed the strongest regional differences, with the largest apical dendrite diameters found in CA3 cells. Statistical analysis of differences in cell morphology across hippocampal subregions is given in Fig. 2g and Supplemental Fig. S1.

### Dendritic axon origins

Cells with dendritic axon origins are very common in the ventral CA1 of the mouse hippocampus, comprising around half of all pyramidal neurons (Thome et al. 2014; Stevens et al. 2024). However, ex vivo investigations targeting dorsal CA1 found much lower numbers (Benavides-Piccione et al. 2020). When we quantified the propensity for this morphology across the whole hippocampus, we found that AcD cells are common across all hippocampal regions (Fig. 3a and b), but with large differences in distribution across all anatomical axes. The dorsal hippocampus featured generally less dendritic axon origins compared to medial and ventral planes. Within individual cutting planes, AcD incidence was significantly larger in the hippocampal CA1 and CA3 areas compared to the subiculum. Interestingly, whereas central CA1 in the dorsal hippocampus shows the lowest incidence of AcD cells, their number reaches the highest levels in the medial and retains high levels in the ventral hippocampus (approximately 50%). We also verified that AcD prevalence decreases from superficial to deep cell layers in ventral CA1 areas. The functional relevance of AcD morphology is likely dependent on the longitudinal electrical resistance that separates the soma from the AIS (Thome et al. 2014; Fekete et al. 2021). We therefore measured the length and thickness of the AcD stem dendrite (Fig. 3c and d). Like the general AcD propensity, the median lengths of stem dendrites were shorter in the dorsal compared to the medial and ventral portions of the hippocampus across CA1 and CA3, while CA3 featured generally longer AcD stem dendrites compared to CA1 and subiculum. AcD stem diameters were similar along the dorsal-ventral axis but show considerable differences between hippocampal regions (Fig. 3d). CA3 showed larger diameters compared to CA1 and in medial and ventral slices while the subiculum featured intermediate levels. Superficial pyramidal cells tended to have longer AcD stems in medial and ventral sections, with highest discrepancies within ventral CA1 (*r*-value: −0.43 in proximal CA1 of ventral slices, Fig. 3e). A minor subpopulation had their AIS arising from the apical dendrite (<1%, data not shown). Profiles of individual cell geometries split by AcD morphology are given in Supplemental Fig. S5.

To test whether these organizational principles extend to humans, we examined CA1 tissue from three newly deceased persons (aged 56 to 76, both sexes) to compare our mouse data to human tissue (Fig. 3f). Proximal cell morphology was visualized using immunostaining with a somatic marker (NeuN) and the same AIS marker used in mouse tissue (βIV-spectrin). Dorsal and ventral sections of the mouse hippocampus correspond to the posterior and anterior portions of the human hippocampus, respectively. In two of three samples, we found larger AcD cell numbers in the ventral/anterior sections, reproducing the pattern of the mouse hippocampus (Fig. 3g).

### Morphological profiles

**Fig. 4:**
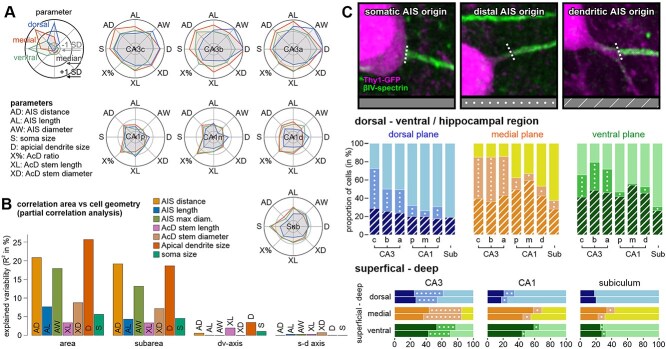
Cell morphology profiles across hippocampal subregions. a) Radar chart plots depicting normalized median values of morphological parameters. Circles show the median of all cells across all regions (middle circle), +/− standard deviation (outer/inner circle). Plots are color coded according to dorsal-ventral axes. b) Partial correlation analysis visualizes how much of the observed variation in cell geometry could be predicted from the location of a given cell. Hippocampal areas and subareas have the most profound impact. c) Classification of cell geometries in cells with somatic AIS origin, distal AIS origin (>5 μm), and AcD morphology. Bars show ratios of all animals and hippocampi pooled. Cells with AcD geometry were most abundant in medial and ventral hippocampal areas and showed generally lower numbers in the subiculum.

Hippocampal subregions exhibited individual profiles of proximal morphologies, in which cells from major hippocampal regions were similar in shape (Fig. 4a). Measures of AIS length, diameter, distance, and somatic/dendritic size were generally smaller in the dorsal hippocampus compared to more ventral planes across all subregions, pointing to a systematic effect modulating the morphology of all principal neurons across the hippocampal longitudinal axis. We used nonparametric pairwise partial correlation analysis to investigate the impact of cell location on proximal cell morphology (Fig. 4b). The hippocampal region had the biggest influence on cell morphology, followed by the dorsal-ventral axis. The depth inside the cell layer had no general effect on any morphological parameter.

For better visualization, pyramidal neurons were separated into three different classes according to their axon origin: Neurons with somatic axon origin, distal axon origin (>5 μm from the soma), and dendritic axon origin (Hofflin et al. 2017). We thus investigated how these subgroups distribute across the hippocampal formation (Fig. 4c). The proportion of these morphologies changed gradually along with the hippocampal subgroups and dorsal to ventral planes. As previously described, AcD cells were common across all regions, but their numbers are lower in the dorsal plane and peak in the medial and ventral regions. Distant axon origins that are not accompanied by AcD morphology were rare in most hippocampal subregions apart from CA3, independent of the respective amount of AcD morphology. In dorsal slices, distant AIS cells were more prevalent in the superficial pyramidal cell layer, whereas their numbers remained stable in medial and ventral hippocampus despite the higher frequency of AcD morphology in superficial layers (see Fig. 3e).

### Interdependencies of proximal cell geometries

**Fig. 5:**
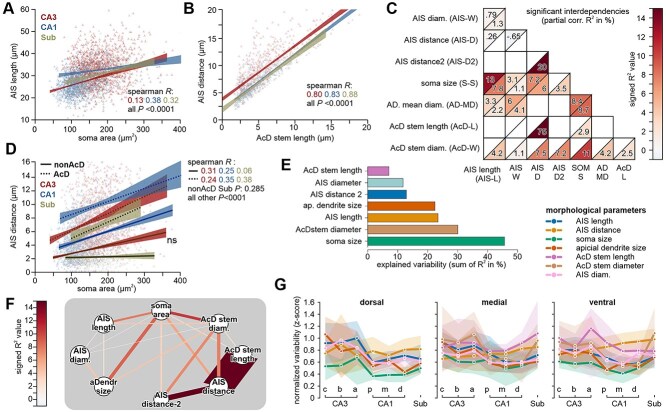
Interdependencies between parameters of cell morphology. a) Scatter plot and linear regression analysis for significant correlations of AIS length with soma size in CA3, CA1, and subiculum. Shaded areas depict 95% confidence intervals. b) Scatter plot and linear regression analysis for significant correlations of AIS stem length with AIS distance in CA3, CA1, and subiculum. c) Partial correlation analysis of proximal cell morphology reveals several statistically significant relationships between soma size and AIS geometry. The length of the AcD stem shows comparably little correlation with the other parameters of cell morphology (R2 values show percentage of predicted variability; partial correlation analysis from all CA1, CA3, and subicular cells controlled for hippocampal area and dorsal/ventral axis. Values for AcD cells are depicted in lower right, for non-AcD cells in upper left corners (3,326 cells from 12 animals). More correlations are given in Supplemental Fig. S6. d) Scatter plot and linear regression analysis for significant correlations of AIS distance vs soma size in CA3, CA1, and subiculum, split between AcD and non-AcD cells. e) The sum R2 for each parameter of cell morphology illustrates how well the variability of each parameter can be predicted from the others (partial correlation analysis). f) Visualization of the strength of correlations with the color code given in C. g) Line plot of the variability of each morphological parameter across the hippocampal formation. Variability is computed as the SD of normalized (Z-scored) values for direct comparison across parameters. Confidence intervals reflect the standard deviation across hippocampi.

Parameters of cell morphology were found to correlate in a compensatory way, leading to overall similar excitability (Hamada et al. 2016). We thus analyzed the interaction of different proximal cell geometries and confirmed established and expected interactions throughout all cell classes, like the positive correlation of cell size with AIS length (Fig. 5a). As expected, AcD stem length correlated strongly with the distance of the AIS, accounting for approximately 78% of the measured variation (*P* < 0.0001, *r* = 0.80, partial correlation controlled for cell location in hippocampal formation; Fig. 5b). We now asked which parameters and interdependencies dominate AIS geometry across the hippocampal formation.

Using partial correlation analysis (including all measured parameters and cell positions), we distilled a general scheme of interdependencies subtracting the effect of anatomical factors (position in dorsal-ventral axis and cell type; Fig. 5c and Supplemental Fig. S6). Cell size correlated generally more often and stronger with the remaining parameters of cell geometry, in particular with AIS length and diameter, as well as apical dendrite diameter. In AcD cells, the diameter of the AcD stem correlated more strongly with other morphological parameters compared to the AcD stem length, apart from the linked parameter of AIS distance. The soma size correlated positively with the length of the AcD stem dendrite. However, soma size generally correlated with AIS distance which, in the case of AcD cells, is tightly correlated with AcD stem length. When we compared linear regression models of AIS distance and soma area in AcD cells vs. non-AcD cells, we found similar correlation coefficients for both groups in CA1 and CA3. Though in AcD cells the AIS were generally located at more distant positions (Fig. 5d). When we limited the analysis of non-AcD cells to the ones with an AIS distances >2 μm, as it is a precondition for AcD cells, we found symmetrical patterns of partial correlations for both cell populations (Supplemental Fig. S6A).

We proceeded with partial correlations to estimate how much variability in a given parameter can be predicted from the data we obtained about the other parameters (Fig. 5e). Because of the dominant intrinsic connection of AIS distance and AcD stem length in AcD cells, we included an alternative definition of AIS distance in this analysis (AIS-distance-2, compare Fig. 3a) which describes the distance from the somatodendritic compartment (including the AcD stem) to the AIS. We found that around 45% of the variability of cell size can be predicted solely from the other measured parameters, with decreasing levels of predictability for AcD stem diameter and AIS length.

Interestingly, the length of AcD stem dendrites was the most independent parameter, indicating that variability in this parameter is only weakly counteracted by proximal cell morphology (Fig. 5f, see also Supplemental Fig. S5 and S6). This indicates that homeostatic compensation of AcD morphology would be less directed to the length of the AcD stem dendrite but rather its diameter, apart from the general and more dominant correlations associated with distal AIS origins. Note that these statistical comparisons are blind to the actual physiological impact that individual parameters might contribute to the function of a given neuron.

We also analyzed the variability of morphological parameters across different locations in hippocampal planes (Fig. 5g). The dorsal axis shows a distinct pattern of variability, with the CA3 region displaying the highest variability across most parameters, with a noticeable decrease in variability compared to CA1. Along the medial axis, variability is more evenly distributed across the hippocampal regions, with a slight increase observed in the subregions. In the ventral axis, variability trends are more dispersed, with no single region showing markedly higher variability. Parameters of AcD morphology were excluded in the dorsal hippocampus due to their lower incidence.

Computer predictions: we used our dataset to predict the variability in signal integration properties that may arise specifically from the proximal cell morphology. We were particularly interested in whether features that may increase neuronal excitability, such as AIS length and distal positions, were compensated by those that reduce excitability, such as larger capacitive sinks through the soma and apical dendrite as shown for other regions (Hamada et al. 2016).

**Fig. 6:**
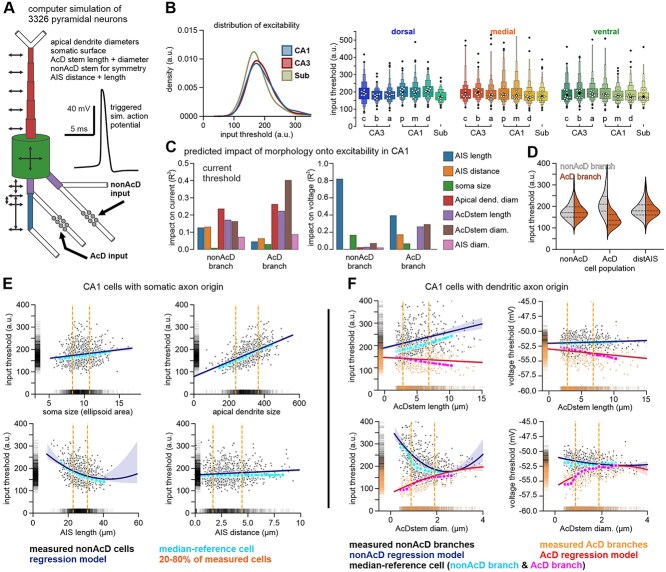
Computer simulation of neuronal excitability using measured cell morphology. a) Cell compartments of a simulated neuron were modified according to manual measurements. Increasing synaptic inputs were simulated either at the non-AcD or AcD type dendritic branch until the cell generated an action potential. For non-AcD cells, the AcD stem length was set to zero. b) Mapping the predicted excitability for all measured cell geometries produced a wide range of input thresholds across all hippocampal subregions. c) Using geometries of all CA1 pyramidal cells, the predicted input thresholds showed a large dependence on apical dendrite and AcD stem diameters, especially for the AcD branch (left panel). Voltage thresholds on the other hand were most tightly connected to AIS length though to a lower degree in AcD branches (right panel). Bars are derived from partial correlation analysis. d) Violin plots show the distribution of input thresholds between AcD and non-AcD branches of cell categories defined in Fig. 4c. AcD morphology generated a considerable shift in input efficiency between the AcD and non-AcD branches of cells with dendritic axon origin. e) Excitability of individual non-AcD cells ordered in relation to parameters of cell morphology (gray) compared to a median-reference cell with static parameters (dotted, median values for all parameters of cell morphology but the ones varied on *x* axis). Linear regression of input efficiencies of cells with full morphology (line) vs median-reference cell shows a strong overlap, illustrating that there is only limited potential for systematic homeostatic compensation. f) The plot of current thresholds (left panels) and voltage thresholds (right panels) vs AcD stem lengths (top) and diameters (bottoms) illustrates the divergence of input efficiencies with increasing AcD morphology (longer and thinner AcD stem) between the AcD branch and non-AcD branches of all CA1 cells in the dataset (dotted). AcD and non-AcD branch of a median-reference cell (see legend for color information, respectively) show a strong overlap with the regression analysis of the full dataset, suggesting limited systematic homeostatic compensation. Second-order polynomial regression was used for AIS length in e), as well as AcD stem diameter in f).

Using a published model of a CA1 pyramidal cell (Hines 1984; Hines et al. 2009; Hodapp et al. 2022), we explored how the natural variability of proximal cell morphologies might translate into a range of different neuronal excitabilities, disregarding other properties such as ion channel densities which were kept constant throughout our experiments. Based on the morphologies of all fully traced pyramidal cells, we constructed individual computer simulations of neurons with synaptic inputs at two basal dendrites (Fig. 6a). This minimalistic input scheme was chosen to allow direct comparison between the AcD and a geometrically balanced non-AcD branch, isolating the impact of axonal origin while maintaining symmetry in electrotonic structure (Supplemental Fig. S7 and S8). We injected stochastic inputs into a simulated non-AcD branch and AcD branch (for AcD neurons) and determined the input threshold at which the cell fired an AP. All cells fired APs within a realistic range of input currents and voltage thresholds (200 to 300 pA; −58 to −50 mV; see also Supplemental Fig. S9). Though medians of neuronal excitability were predicted to be different between different brain regions and dorsal to ventral axes, their distributions were strongly overlapping (Fig. 6b). We then evaluated the contribution of each geometric parameter to calculated input efficiencies in all CA1 pyramidal neurons (Fig. 6c). Parameters of membrane size such as apical dendrite diameter and AcD stem diameter (but not soma size) had a strong predictive power for input efficiency. The voltage threshold, measured at the soma, showed the highest correlation with AIS length. It has been established that neurons with and without AcD morphology do not differ in their gross neuronal excitability, but in how they integrate inputs from the AcD and non-AcD branches (Thome et al. 2014; Hamada et al. 2016; Hodapp et al. 2022). Our model cell was equipped with two basal dendritic branches which were modified according to our measurements. Simulated input at either branch of non-AcD cells resulted in equal firing thresholds for both branches (left plot in Fig. 6d). Dendritic branches of non-AcD cells with distal AIS origins featured higher input thresholds, but with a generally similar range of input efficiencies. In the case of AcD cells, both branches diverged in their ability to trigger APs, which broadened the range of input efficiencies without changing the size or ion channel composition of each dendrite. Note that a proximal section of the second non-AcD branch was always modified in length and diameter to keep symmetry between branches intact.

While our simulation predicted a large variability in input efficiencies driven by proximal cell geometry, it could not establish whether individual geometries may act as homeostatic modulators, driving the cells back to a defined band of allowed excitability, or as agents of diversification that boost variability in our simulation. To tackle this question, we defined a stereotypic median CA1 pyramidal cell in which soma size, apical dendrite diameters, as well as AIS length, diameter, and position were kept at the medium value found in our dataset. We then modified only one of those parameters in the range of the 1^th^ to 99^th^ percentile of our dataset (Fig. 6e, light blue dots). This experiment illustrates how our computer model predicts the impact of individual geometric cell parameters in general when all other parameters remain constant. We then compared the predicted input thresholds of this median-reference cell with ones of fully measured geometries (Fig. 6e, black dots). Applying simple regression models revealed that input efficiencies of cells with real geometries showed the same trajectory with eg AIS length as the stereotypic CA1 cell. Other correlations of cell geometries also followed the standard cell tightly, which lacks any potential homeostatic compensation (see also Supplemental Fig. S3G for AIS diameter). This was also true in AcD neurons for which we measured input thresholds individually for the AcD and non-AcD branch of each neuron (Fig. 6f). To further dissect the spatial dynamics of excitability along the AIS, we performed targeted simulations with current injections at the soma, proximal AIS, and distal AIS, while varying AIS length, distance, diameter, soma size, and AcD stem geometry as in Fig. 6e and f. The distal AIS consistently exhibits the highest excitability, particularly in cells with longer or thinner AIS (Supplemental Fig. S9A and B). In addition, we quantified the delay between stimulus onset and AP initiation and found that temporal dynamics of spike initiation were particularly sensitive for AIS length and position if current was injected at the distal AIS, the presumed initiation site of most APs (Supplemental Fig. S9C).

We thus propose that the variability of AIS geometry has a dual impact on neuronal excitability. The interplay between AIS distance and AIS length on the one hand may compensate for somatic size and other anatomical and molecular mechanisms (see Fig. 5). On the other hand, AIS geometry also increases neuronal diversity by enlarging the variability of neuronal excitability. The second case was dominant in our computer model although it is likely that other internal factors (eg ion channel composition, synaptic organization) as well as external factors (volume, position, and composition of excitatory and inhibitory drives) play a major role in setting the stage for the establishment and modulation of proximal cell geometries.

## Discussion

Understanding the structural variability of the AIS in hippocampal neurons is essential for uncovering how neuronal excitability and AP generation are regulated. In this study, we conducted a comprehensive analysis of AIS geometry across the murine hippocampus, examining how variations in AIS geometry, position, and proximal cell morphology contribute to functional diversity. By mapping these features along the dorsal/ventral, superficial/deep, and proximal/distal axes, we identified region-specific patterns in AIS morphology, with dorsal cells showing the most pronounced differences, including smaller somata, shorter and more distal AIS, and fewer AcD geometries. AIS lengths and distances peaked in intermediate CA3, with ~35 μm AIS length and over 80% of neurons with an AIS originating >5 μm from the soma, in contrast to adjacent CA1 neurons where AIS were ~10 μm shorter. Dendritic axon origins were most frequent in the intermediate and ventral hippocampus, a trend seen across all subregions. These findings are consistent with previous studies that have reported regional differences in hippocampal neuron morphology (Malik et al. 2016; Mehder et al. 2020; Hodapp et al. 2022). Despite these regional trends, we have observed considerable overlap in AIS geometry across subregions, which suggests that individual variability exceeds interregional differences, highlighting the broad morphometric diversity of hippocampal neurons that may be obscured by population averages (Grubb and Burrone 2010). The difference within each neuronal subpopulation was greater than the differences *between* them, rendering the main hippocampal regions more similar than dissimilar.

The observed diversity in AIS geometry may in fact serve multiple, at times opposing, roles. It may reflect multiple structural solutions for maintaining excitability homeostasis, while also expanding the parameter space for baseline intrinsic excitability and modes of input integration. At the same time, it may also allow for a differentially tuned susceptibility to AIS plasticity, thereby supporting flexible modulation of neuronal excitability and firing precision (Jamann et al. 2021; Rotterman et al. 2021). In ventral CA3 neurons, for example, longer and more distal AIS may compensate for larger somatodendritic compartments, ensuring rapid responses to synaptic input. Such morphology-dependent tuning is consistent with prior studies showing activity-driven shifts in AIS length and position (Grubb and Burrone 2010; Kuba 2012; Jamann et al. 2021). While AIS plasticity has been widely modeled and linked to excitability changes (Goethals and Brette 2020), the extent to which it reflects homeostatic regulation versus morphological variability remains unclear (Rotterman et al. 2021). In our dataset, interactions of proximal geometry explained between 7% (AcD stem length) up to 45% (soma area) of the variance in cell morphology (Fig. 5e). Since we could not infer to which degree these correlations attribute to homeostasis or increase the functional variability of neurons, we utilized computer simulations to investigate which role was dominant, though we disregarded other parameters such as distinct ion channel distributions and cell geometries. We received a wide range of predicted excitabilities, reminiscent of electrophysiological data (Booker et al. 2020; Thome et al. 2025), but found little evidence of homeostatic balancing (Fig. 6e and 6f). Rather, our model suggests that AIS diversity is one of several factors contributing to the wide range of neuronal excitability observed in electrophysiological recordings. While such a variability is essential for stable network activity and information coding function (Pena and Rotstein 2022), specific AIS geometries may be more susceptible to plasticity, further supporting dynamic regulation of neuronal output.

Our study confirms previous reports of dendritic axon origins in CA1 pyramidal neurons of both mice and humans, though reported frequencies vary widely between studies. While we consistently found AcD morphology in approximately 50% of murine CA1 neurons (Thome et al. 2014; Hodapp et al. 2022; Stevens et al. 2024), others reported only about 20% (Benavides-Piccione et al. 2024), likely due to methodological differences such as cutting plane orientation. In human CA1, AcD morphology has been reported in up to 44% of neurons without separating anterior and posterior regions. Our own human dataset hints at a conserved dorsal/posterior or ventral/anterior pattern between species (Fig. 3g). However, larger sampling numbers are needed to determine whether this proportion is representative or incidental. Regarding homeostasis, AcD neurons in our dataset did not deviate from the general correlations observed in non-AcD neurons, but showed patterns similar to cells with distal AIS (Fig. 5, Supplemental Fig. S6). This suggests that the presence of a dendritic axon origin is not structurally compensated in a way that offsets its influence on proximal morphology. A previous study in rat cortical neurons reported that AcD cells exhibit narrower apical dendrites to normalize AP waveforms (Hamada et al. 2016), for which we found only little evidence in hippocampal pyramidal neurons (Supplemental Fig. S6). AcD stem length showed minimal correlation with overall cell geometry, suggesting that it is not a major target of functional homeostasis, which were furthermore surpassed by the correlations of AIS distance, a parameter that is inherently linked to AcD stem length. Additionally, although AcD diameter correlated strongly with multiple aspects of cell morphology, it scaled only weakly with stem length, making it an unlikely candidate for offsetting AcD-specific excitability changes. Together, these findings reinforce the notion that AcD morphology is largely tolerated rather than structurally counterbalanced. We can only hypothesize about the functional implications of the higher AcD prevalence in ventral compared to dorsal CA1. Ventral CA1 pyramidal neurons exhibit higher intrinsic excitability despite reduced dendritic complexity (Dougherty et al. 2012; Malik et al. 2016; Milior et al. 2016), potentially favoring the emergence of “privileged” dendritic branches that integrate inputs of particularly salience. This region is also associated with emotional aspects of memory processing which may involve stronger interhemispheric connectivity, in turn associated with AcD cells in ventral CA1 (Stevens et al. 2024).

### Limitations of the study

The depth of our anatomical segmentation limited the number of animals and dorsal sections were obtained from separate individuals due to slicing constraints, complicating comparisons both within and across animals, including potential sex differences. Additionally, the use of the Thy1-GFP mouse line, while common, introduces potential bias towards neuronal subpopulations and did not permit the full analysis of CA2 neurons, which are usually Thy1-GFP-negative. However, the distribution of AcD cells in our dataset is consistent with previous studies using nongenetic labeling (Thome et al. 2014; Benavides-Piccione et al. 2020; Hodapp et al. 2022) and AIS lengths were comparable between Thy1-GFP positive and negative cells of the same slices (verified in CA1 and CA3), supporting the representativeness of our sampled population with respect to proximal cell geometry. Our simplified computational model does not capture the full biological complexity of neuronal excitability. In particular, it lacks the diversity of dendritic arborization, ion channel distributions, and dynamic synaptic input patterns that shape neuronal firing. The two-dendrite input model simplifies synaptic integration and omits potential interactions between inputs across different branches. We decided to utilize this simplified model, because experimental validation of AIS function also presents significant challenges. Functional assessments rely largely on somatic patch-clamp recordings and current injections, which have revealed correlations between AIS geometry and excitability primarily in paradigms of AIS plasticity (Grubb and Burrone 2010; Kuba et al. 2010; Jamann et al. 2021), but less consistently in unperturbed neurons (Hamada et al. 2016; Stoler and Fleidervish 2016; Moubarak et al. 2019; Thome et al. 2025). Measuring excitability solely through current and voltage thresholds may not fully capture the functional roles of the AIS. For example, neurons with disrupted AIS cytoskeletons can still generate APs but lose responsiveness to high-frequency input (Revah et al. 2019). Such recordings are further confounded by technical artifacts such as slicing damage, somatic swelling, and AIS disassembly, all of which can obscure or mask morphology-dependent effects. Given these constraints, our computational approach offers a controlled framework to isolate and systematically evaluate the influence of the AIS, while it does not replicate full physiological complexity.

Given the translational relevance of AIS morphology, future research should investigate how its variability relates to neurological disease and aging. Alterations in AIS structure have been associated with conditions such as epilepsy, schizophrenia, and autism spectrum disorder (Ferreira et al. 2008; Wimmer et al. 2010; Buffington and Rasband 2011; Hsu et al. 2014; Huang and Rasband 2018; Fujitani et al. 2021; Usui et al. 2022; Garrido 2023). Extending our findings to human tissue across a range of pathological states will be essential for deepening our understanding of AIS organization in the so far understudied healthy human brain, but also for identifying how AIS geometry adapts, or maladapts, in the context of neurological disease. We believe such knowledge forms a crucial foundation for unraveling disease mechanisms.

## Supplementary Material

AISmorphology_supplementary_material_v23_bhaf297

datarespository_v03_bhaf297

## Data Availability

The analysis provided in this publication does by no means exploit the full depth of the collected dataset, especially regarding patterns specific to neuronal subpopulations as well as CA2 and hilus. The authors invite all readers to use the provided dataset for their investigation. The full dataset and custom Python code used for analysis are provided in the supplemental files. The core of the cell model is available through: https://doi.org/10.11588/data/JWLFFZ and modifications are summarized in Supplemental File 2 “new_code.”
