# Supplementary material for: Diversity of axon initial segment geometry in the mouse hippocampus and its predicted influence on neuronal excitability
Source: Cereb Cortex. 2025 Dec 9;35(12):bhaf297. doi: 10.1093/cercor/bhaf297 (PMC12687872; doi:10.1093/cercor/bhaf297)
Supplement: AISmorphology_supplementary_material_v23_bhaf297 [file aismorphology_supplementary_material_v23_bhaf297.docx]

**SUPPLEMENTAL MATERIAL**

**Diversity of Axon Initial Segment Geometry in the Mouse Hippocampus and Its Predicted Influence on Neuronal Excitability**

**Nikolas Andreas Stevens^1^, Maximilian Achilles^2,3^, Juri Monath^2,3^, Rupert Langer^,3,4^, Maren Engelhardt^2,3^, Martin Both^1,+^, Christian Thome^1,2,3,+,^***

*^1^ Institute of Physiology and Pathophysiology, Heidelberg University, Heidelberg, Baden-Württemberg, Germany*

*^2^ Institute of Anatomy and Cell Biology, Johannes Kepler University Linz, Linz, Upper Austria, Austria*

*^3^ Clinical Research Institute for Neurosciences, Johannes Kepler University Linz, Linz, Upper Austria, Austria*

*^4^ Department of Pathology and Molecular Pathology, Kepler University Hospital Linz, Linz, Upper Austria, Austria*

*+ Equal contribution as senior authors*

** Corresponding author*

*address: Institute of Anatomy and Cell Biology, Krankenhausstrasse 5, 4020 Linz, Austria;*

*email: christian.thome@jku.at; phone: +4373224688905)*

**Figure S1**

**
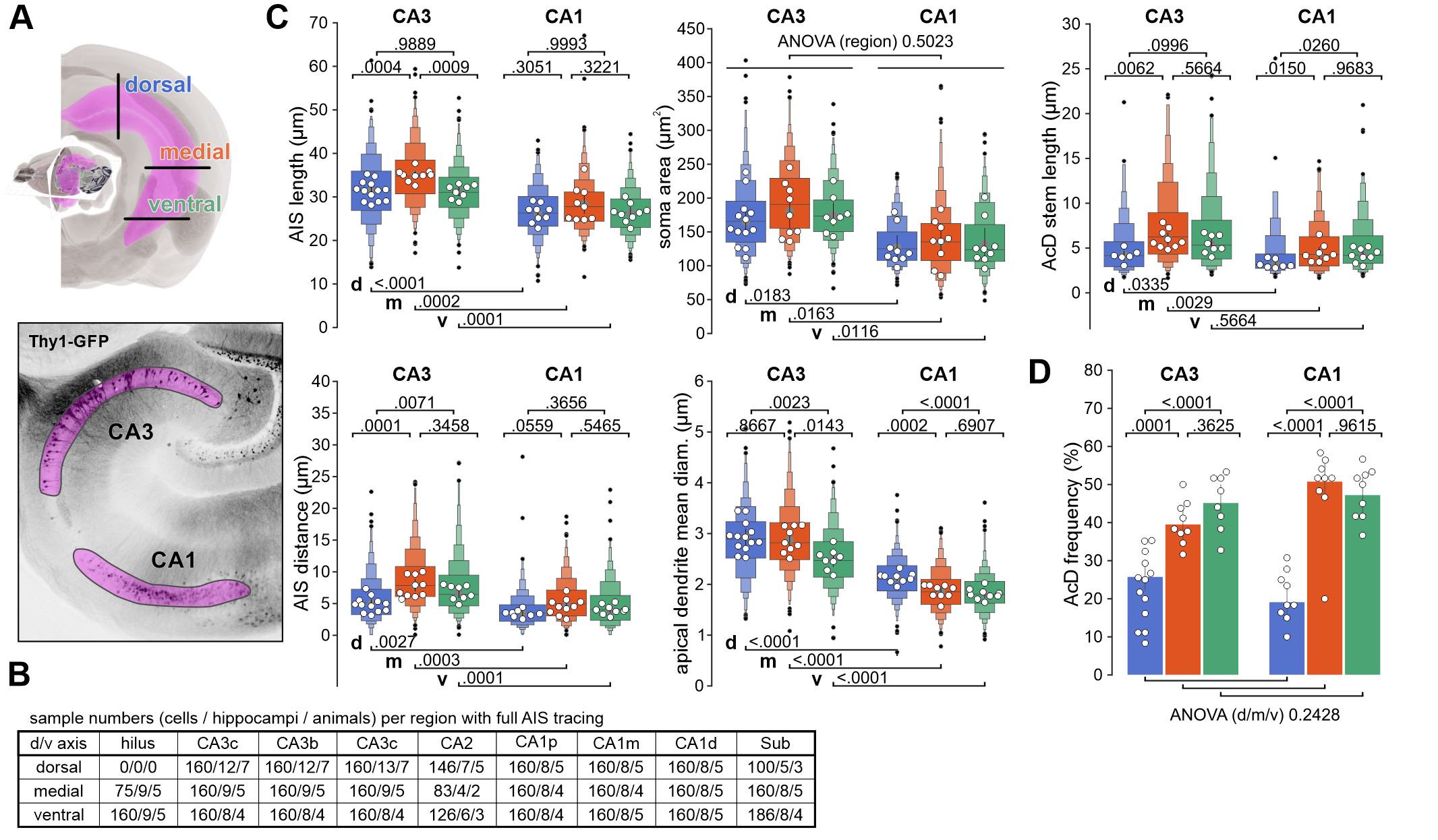
**

**Supplemental Figure S1: Comparison of proximal cell parameters between hippocampal regions in different cutting planes along the dorsal-ventral axis. (A)** Scheme of murine hippocampus (Allen Brain Explorer) illustrating color code of hippocampal sections used in this study (top panel). Bottom panel: Confocal image of Thy1-GFP signal in ventral slice. CA1 and CA3 areas are marked by magenta areas. **(B)** Sample numbers used throughout the study divided by cells, hippocampi, animals, and regions. **(C)** Extended box plots (Letter-value plots, boxenplots in Python’s seaborn package) show distribution of AIS length, AIS distances, soma area, and mean apical dendrite diameter between area CA3 and CA1. White dots depict median values of the region for each hippocampus. Grey dots and lines depict mean value of all hippocampi pooled with 95% confidence interval. The black dots depict outliers as defined by data points falling beyond the extended ranges determined by the letter-value plot method. Specifically, these ranges are dynamically calculated based on the interquartile range (IQR) at progressively smaller proportions of the data distribution, following the 'k_depth' parameter set to 'trustworthy.' Outliers represent values that deviate significantly from the main distribution. Statistical differences were determined by performing an ANOVA to test for differences along the dorsal-to-ventral axis or between hippocampal regions (CA3 and CA1). In cases where significantly different distributions were identified, a Tukey post-hoc test was conducted to determine pairwise differences. *P*-values are indicated above the respective boxes in the figure. **(D)** Barplots depicting the mean percentage of dendritic axon origins across hippocampal regions and across different cutting planes depicted in (A).

**Figure S2**

**
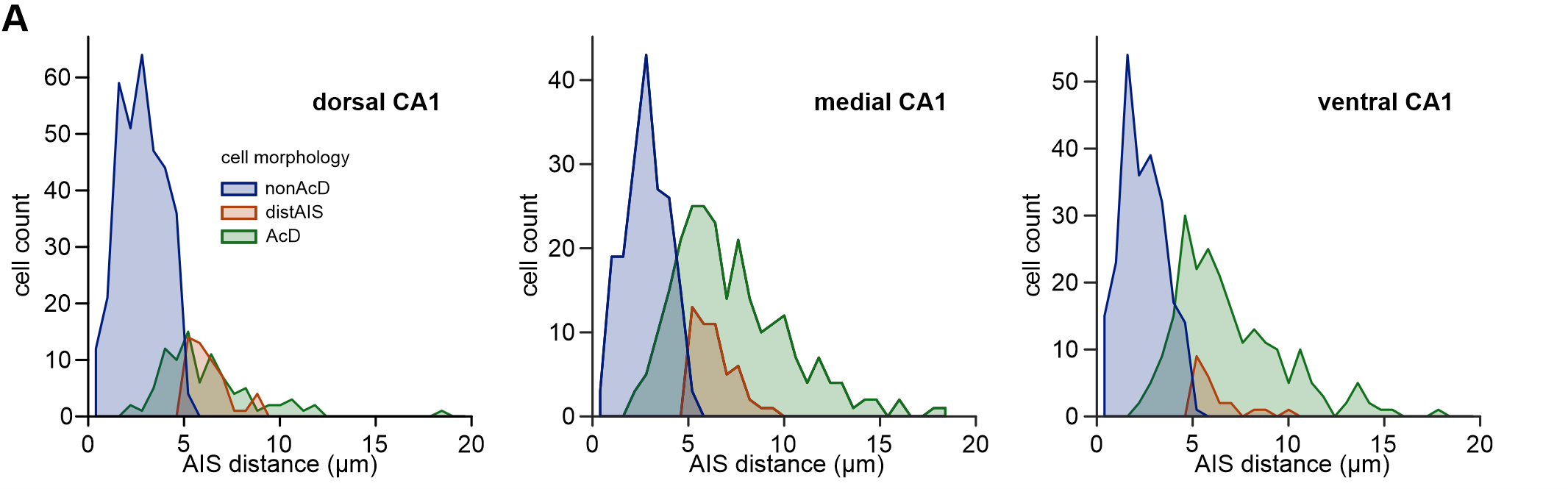
**

**Supplemental Figure S2: Distribution of AIS distances in CA1 pyramidal neurons. (A)** Histograms show distribution of AIS distances in CA1 pyramidal cells separated by AIS origin. In contrast to previous reports (Hodapp et al. 2022), we found no clear bimodal distribution of AIS distances in CA1 pyramidal neurons.

**Figure S3**

**
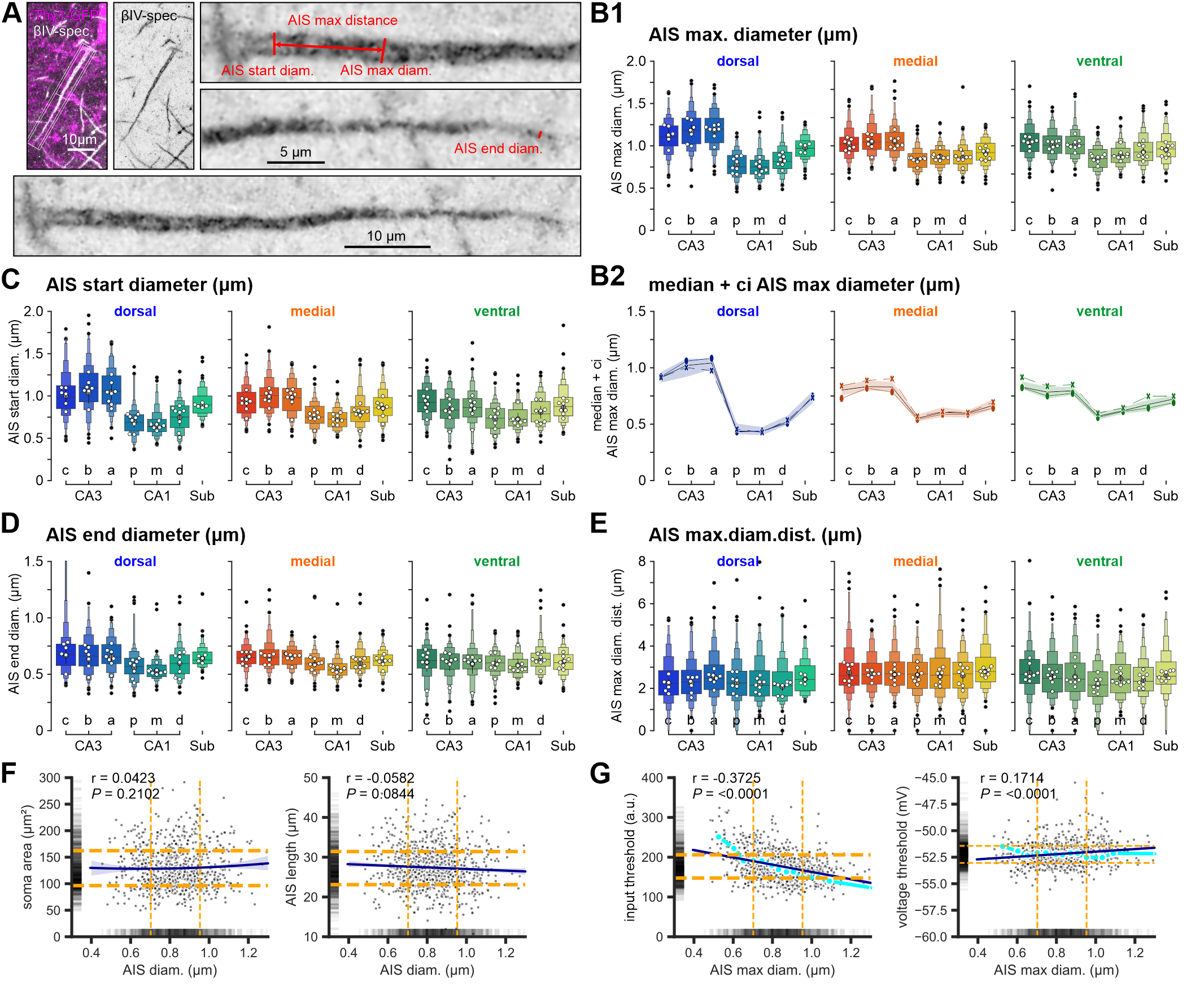
**

**Supplemental Figure S3. AIS diameters across hippocampal subregions. (A)** Confocal image showing intrinsic GFP (magenta) and βIV-spectrin-labeled AIS (white). Red lines indicate measurement points for AIS diameter: start, end, and point of maximum thickness. **(B1)** Extended boxplots of maximum AIS diameters across the cell bands of dorsal (blue), medial (orange), and ventral (green) hippocampus. White lines indicate medians, grey bars show mean of medians ± SD, and black dots represent computed outliers (some outliers <10 omitted for clarity; see Methods). **(B2)** Median maximum AIS diameters by hippocampal region and cellular depth (superficial: x, deep: o). **(C–E)** Extended boxplots of AIS start (C), end (D), and distance from AIS start to maximum diameter (E). **(F)** Scatter plots showing the relationship between maximum AIS diameter and soma area (left) or AIS length (right) in CA1 pyramidal cells. No significant correlations were observed. **(G)** Scatter plots from computational models showing effects of AIS diameter on input threshold (left) and voltage threshold (right). Larger AIS diameters required less input to spike, but voltage threshold remained largely unaffected. Orange lines mark the 20th and 80th percentiles; color coding as in Figure 6E.

**Figure S4**

**
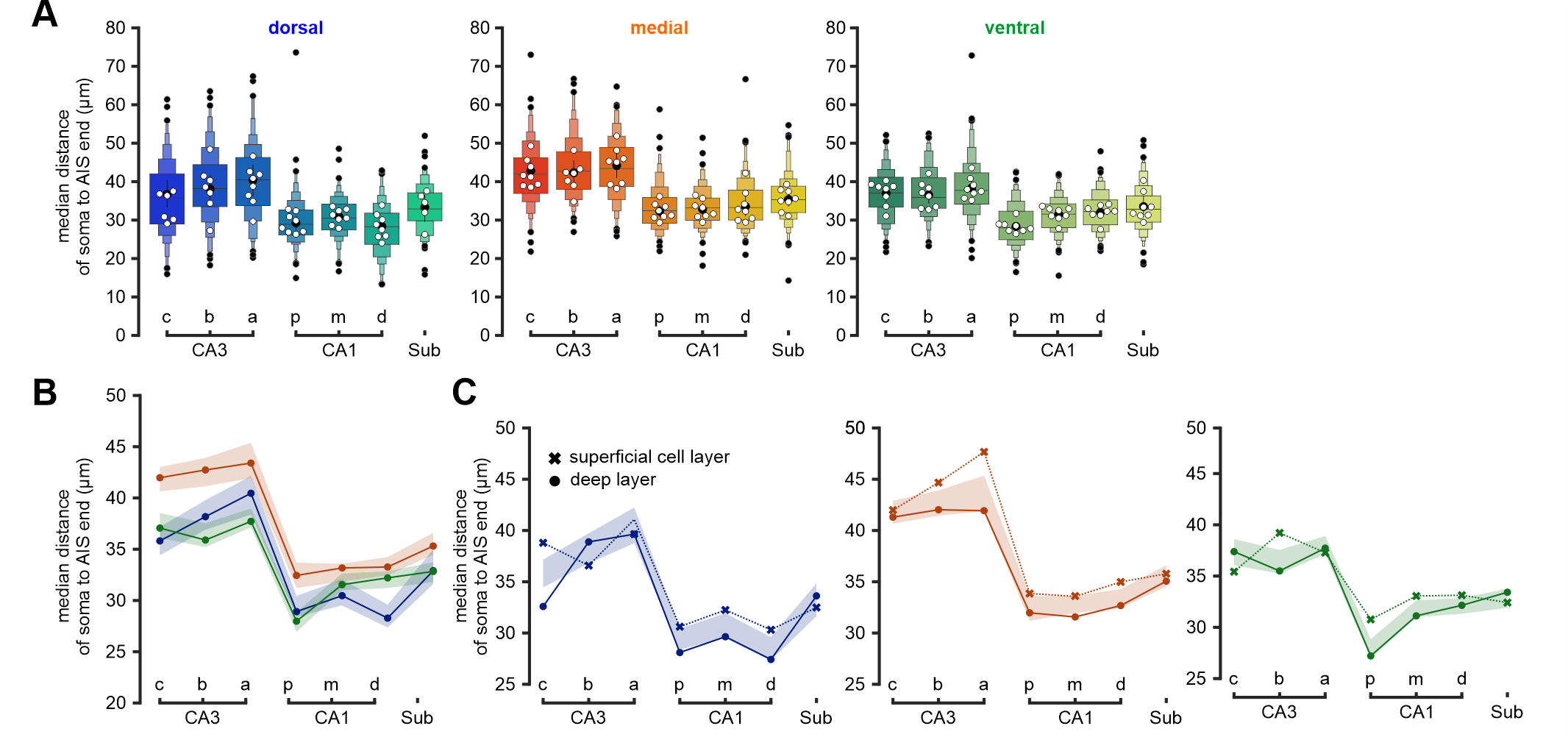
**

**Supplemental Figure S4: Comparison of the distances between soma and the end of the AIS label via βIV-spectrin between hippocampal subregions in different cutting planes along the dorsal-ventral axis. (A)** Extended box plots designed as described in Figure S1C. **(B)** Line plots show the median distance of the end of the AIS fluorescence signal as in (A) for better comparison between dorsal, medial, and ventral axes. **(C)** Line plots show the median distance of the end of the AIS fluorescence signal split according to dorsal-ventral axes and further divided by the cell position within the pyramidal cell band (x: superficial, o: deep).

**Figure S5**


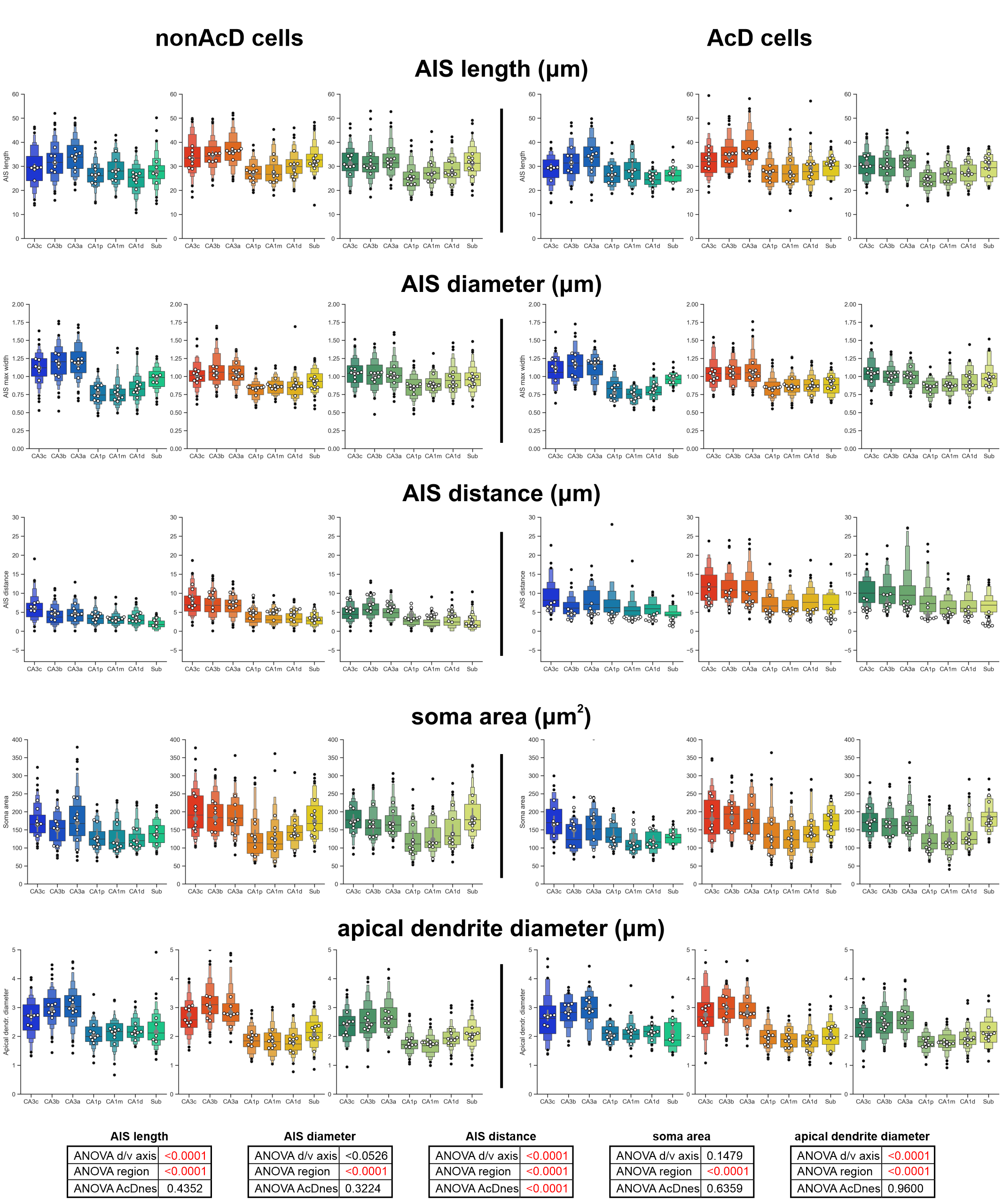


**Supplemental Figure S5. Influence of AIS position on proximal cell morphology.** AIS geometry parameters separated by axon origin (nonAcD vs AcD cells). Bottom panels display results from a 3-way ANOVA testing the effects of AcD morphology on each geometric parameter, controlling for hippocampal region and subfield. No systematic differences in proximal morphology were observed between AcD and nonAcD cells, except for AIS-to-soma distance, an inherent feature of AcD cells. Data from 3.326 cells across 12 animals.

**Figure S6**


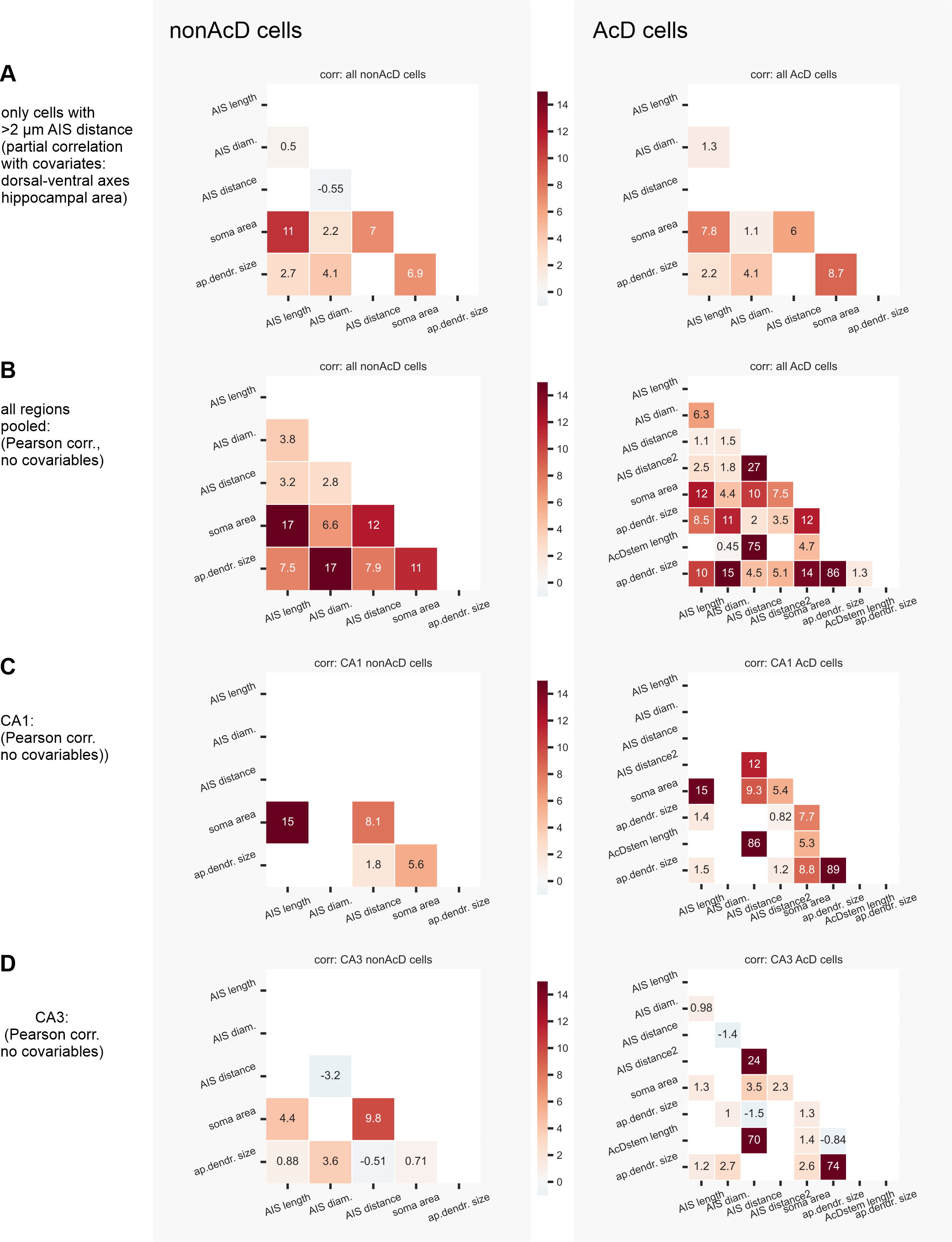


**Supplemental Figure S6. Partial and Pearson correlation analysis of proximal cell morphology reveal several significant relationships.** Boxed R² values indicate the proportion of explained variance. Analyses were performed for different subsets of cells. **(A)** Only cells with AIS distances >2 µm were included to obtain symmetry with AcD cells. The analysis was a partial correlation using the cutting plane and main hippocampal regions as co-variates. **(B-D)** The remaining rows used regular bivariate Pearson correlations on either all pyramidal cells pooled (CA1, CA3, subiculum; **B**), only CA1 pyramidal cells (**C**), and only CA3 pyramidal cells **(D)**. Analysis was performed on cells further subdivided by axon origin: somatic nonAcD (left panels) and dendritic AIS origin (right panels). Total dataset: 3.326 cells from 12 animals (see subsets in Supplemental Figure S1B).

**Figure S7**


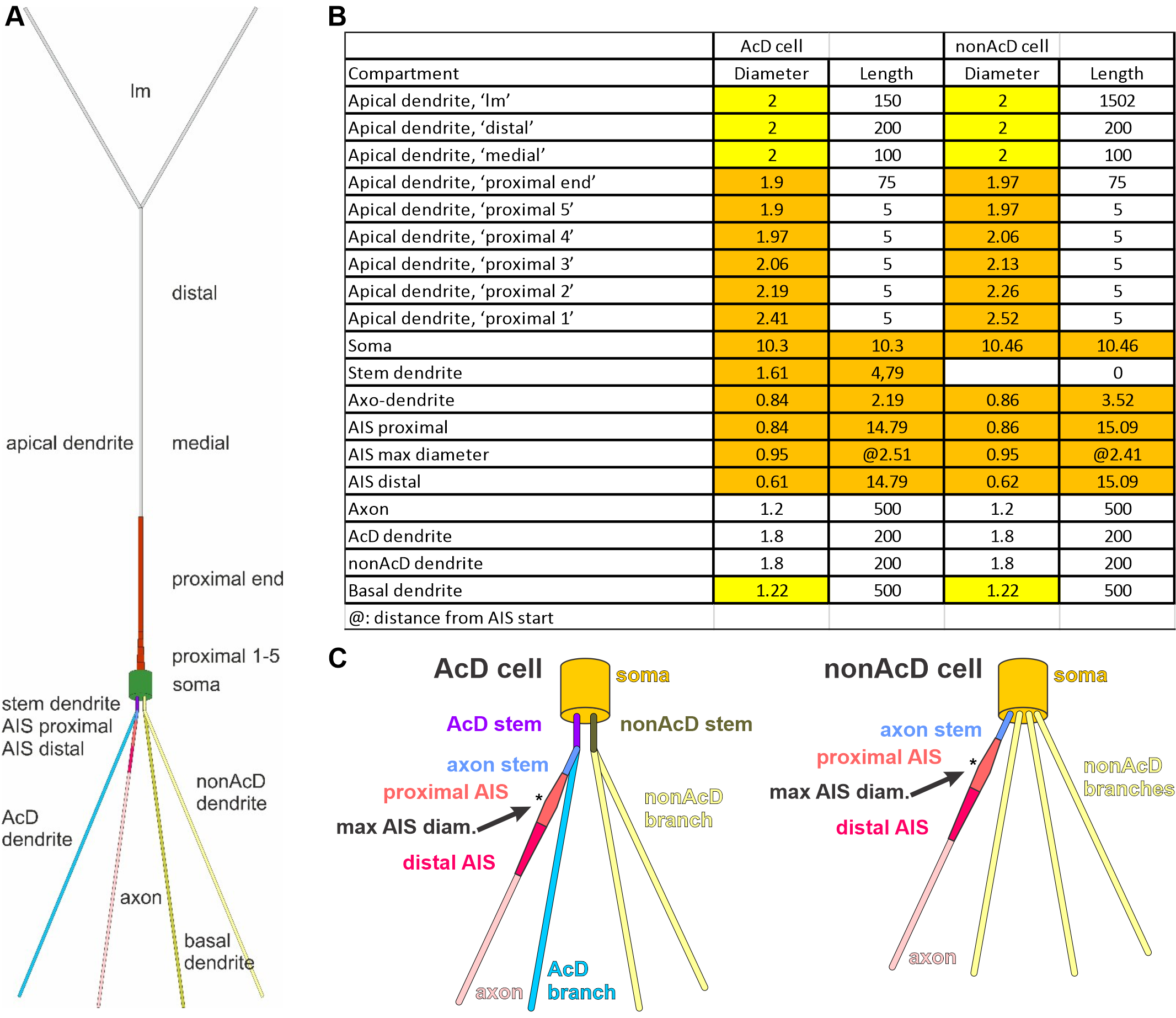


**Supplemental Figure S7. Morphology of the model cell used in simulations.** Adapted from Hodapp et al., 2022. **(A)** Schematic of neuronal compartments and their relative proportions. **(B)** Summary of compartment lengths, diameters, and distances. **(C)** Detailed view of the proximal morphology in AcD (left) and nonAcD (right) model variants. In AcD cells, the nonAcD stem was adjusted to match the neighboring AcD stem, ensuring electrotonic symmetry.

**Figure S8**


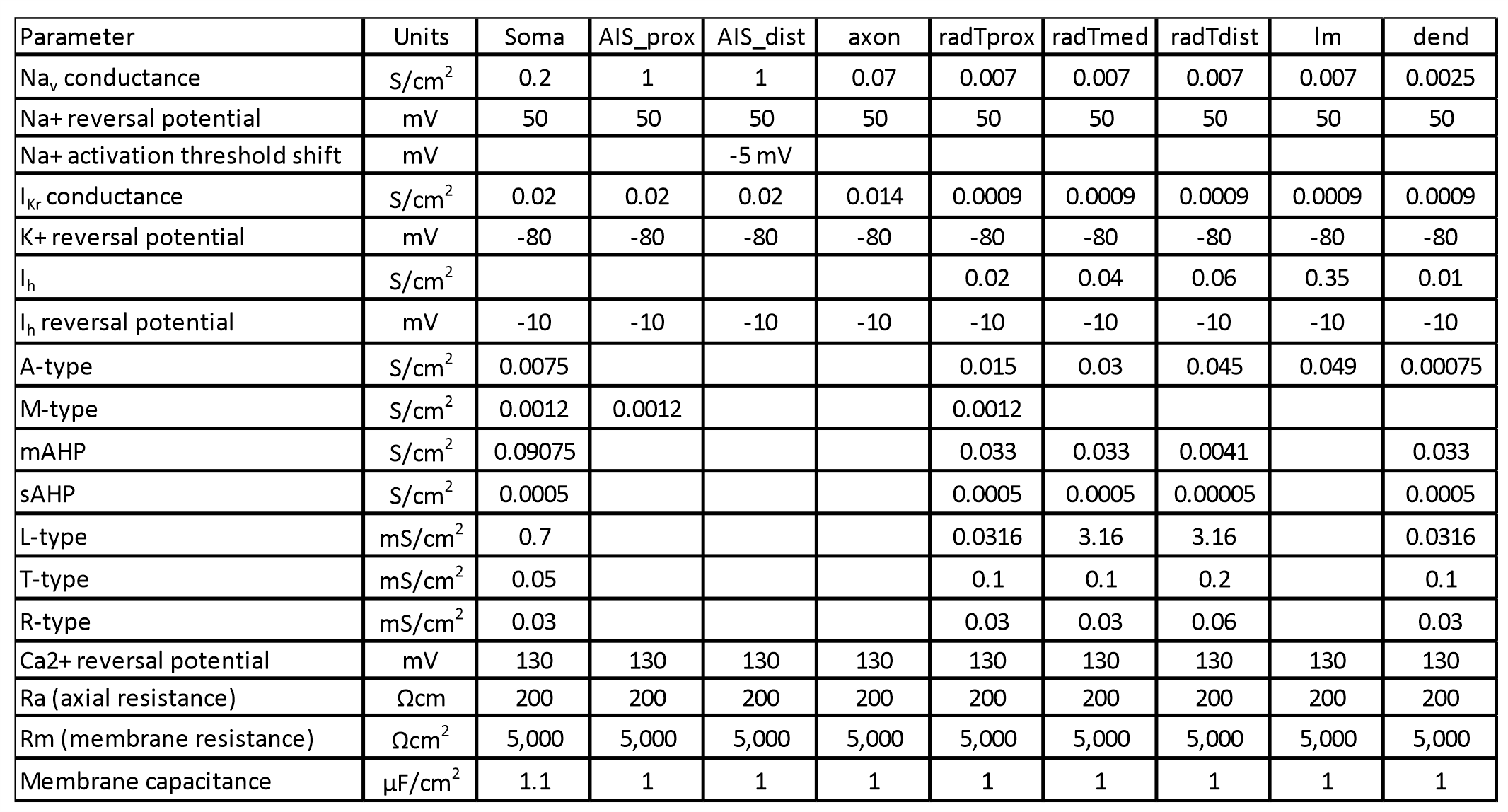


**Supplemental Figure S8. Electrotonic parameters used to model cellular compartments.** Summary of passive and active membrane properties used in the simulations. All values were adopted without modification from Hodapp et al., 2022.

**Figure S9**


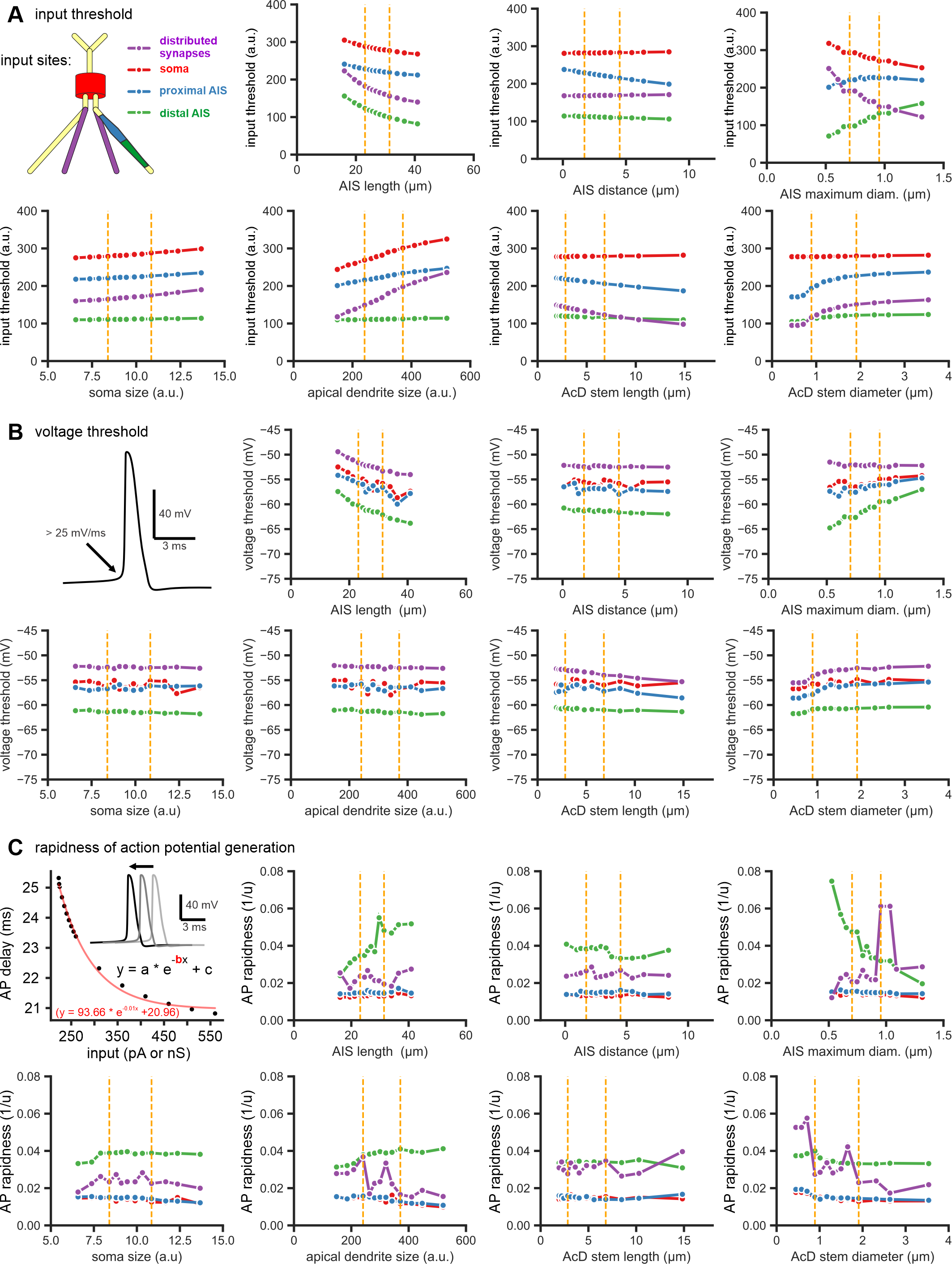


**Supplemental Figure S9: Predicted effects of proximal cell morphology on neuronal excitability across stimulation paradigms.** We used computational modeling to assess input threshold **(A)**, voltage threshold **(B)**, and action potential rapidness **(C)** following synaptic input (purple, in a.u.) and current injection (in pA) into the soma (red), proximal AIS (blue), or distal AIS (green). All models used the median geometry of CA1 pyramidal cells, except for the varied parameter in each condition. At threshold, most action potentials occurred near the end of the stimulus. Their onset latency then decreased with increasing input strength (top left panel in C). Rapidness was quantified by the exponential slope (b) of the input-output relationship. AIS length and maximum diameter were the primary determinants of current and voltage thresholds. Apical dendrite diameter strongly influenced current thresholds for inputs arriving close or passing through the somatic membrane but had minimal impact on inputs at the distal AIS. Distal AIS stimulation consistently yielded lower thresholds and higher rapidness, with AIS length and diameter being the strongest predictors.

**References:**

Hodapp A, Kaiser ME, Thome C, Ding L, Rozov A, Klumpp M, Stevens N, Stingl M, Sackmann T, Lehmann N, Draguhn A, Burgalossi A, Engelhardt M, Both M. 2022. Dendritic axon origin enables information gating by perisomatic inhibition in pyramidal neurons. Science. 377(6613):1448-1452. <https://www.ncbi.nlm.nih.gov/pubmed/36137045>. doi:10.1126/science.abj1861.
